# Comparison of gene-by-gene and genome-wide short nucleotide sequence-based approaches to define the global population structure of Streptococcus pneumoniae

**DOI:** 10.1099/mgen.0.001278

**Published:** 2024-08-28

**Authors:** Alannah C. King, Narender Kumar, Kate C. Mellor, Paulina A. Hawkins, Lesley McGee, Nicholas J. Croucher, Stephen D. Bentley, John A. Lees, Stephanie W. Lo

**Affiliations:** 1 Parasites and Microbes, The Wellcome Sanger Institute, Wellcome Genome Campus, Hinxton, Cambridge, UK; 2 Rollins School Public Health, Emory University, Atlanta, GA, USA; 3 Emory Global Health Institute, Emory University, Atlanta, GA, USA; 4 MRC Centre for Global Infectious Disease Analysis, Department of Infectious Disease Epidemiology, Imperial College London, London, UK; 5 EMBL-EBI, Wellcome Genome Campus, Hinxton, Cambridge, UK; 6 Milner Centre for Evolution, Department of Life Sciences, University of Bath, Bath, UK

**Keywords:** epidemiology, MLST, population structure, pneumococcal genomics, *Streptococcus pneumoniae*, whole genome sequencing

## Abstract

Defining the population structure of a pathogen is a key part of epidemiology, as genomically related isolates are likely to share key clinical features such as antimicrobial resistance profiles and invasiveness. Multiple different methods are currently used to cluster together closely related genomes, potentially leading to inconsistency between studies. Here, we use a global dataset of 26 306 *Streptococcus pneumoniae* genomes to compare four clustering methods: gene-by-gene seven-locus MLST, core genome MLST (cgMLST)-based hierarchical clustering (HierCC) assignments, life identification number (LIN) barcoding and k-mer-based PopPUNK clustering (known as GPSCs in this species). We compare the clustering results with phylogenetic and pan-genome analyses to assess their relationship with genome diversity and evolution, as we would expect a good clustering method to form a single monophyletic cluster that has high within-cluster similarity of genomic content. We show that the four methods are generally able to accurately reflect the population structure based on these metrics and that the methods were broadly consistent with each other. We investigated further to study the discrepancies in clusters. The greatest concordance was seen between LIN barcoding and HierCC (adjusted mutual information score=0.950), which was expected given that both methods utilize cgMLST, but have different methods for defining an individual cluster and different core genome schema. However, the existence of differences between the two methods shows that the selection of a core genome schema can introduce inconsistencies between studies. GPSC and HierCC assignments were also highly concordant (AMI=0.946), showing that k-mer-based methods which use the whole genome and do not require the careful selection of a core genome schema are just as effective at representing the population structure. Additionally, where there were differences in clustering between these methods, this could be explained by differences in the accessory genome that were not identified in cgMLST. We conclude that for *S. pneumoniae*, standardized and stable nomenclature is important as the number of genomes available expands. Furthermore, the research community should transition away from seven-locus MLST, whilst cgMLST, GPSC and LIN assignments should be used more widely. However, to allow for easy comparison between studies and to make previous literature relevant, the reporting of multiple clustering names should be standardized within the research.

## Data Summary

Genome sequences are deposited in the European Nucleotide Archive, and accession numbers can be found in the supplementary data file (Data S1, avilable in the online version of this article). Metadata of the pneumococcal isolates in this study have been submitted as a supplementary file (Data S1, available in the online version of this article) and are also available on the Monocle Database available at https://data.monocle.sanger.ac.uk/. The authors confirm that all supporting data, code and protocols have been provided within the article or through supplementary data files.

**Impact Statement**

Using a global dataset of *Streptococcus pneumoniae* genomes allows us to thoroughly observe and analyse discrepancies that occur during the clustering of pneumococcal genomes. Whilst all methods in this study are used to cluster *S. pneumoniae* genomes, no study has yet thoroughly compared the clustering results and discrepancies. This work summarizes the strengths and weaknesses of the different methods and highlights the need for consistency between studies.

## Introduction

*Streptococcus pneumoniae* (or pneumococcus) is a clinically important human pathogen responsible for a range of infectious diseases, including otitis media, pneumonia and meningitis. It was estimated to have caused 829 000 (95 % uncertainty intervals 682 000–1 010 000) deaths worldwide in 2019 [1]. The polysaccharide capsule surrounding the cell of the pneumococcus is the basis of a traditional typing scheme, which has been used to separate isolates into groups (i.e. serotypes) [2]. Although the pneumococcal capsule is the major virulence factor and vaccine target, the disease potential [3], transmissibility [4], antimicrobial resistance [3] and responses to vaccine-induced protection [5] are also related to the genetic composition of the strain [3]. Therefore, defining the population structure of pneumococci lays the foundation for epidemiological investigation [4,6] and assessing the impact of clinical interventions, such as vaccine implementation and antimicrobial use for prophylaxis or treatment of disease [7].

Due to high levels of recombination, defining the population structure of pneumococci is particularly challenging. Since 1998, MLST has been widely used to capture the genetic variation of seven housekeeping genes (*aro*E, *gdh, gki*, *rec*P, *spi*, *xpt* and *ddl*) to classify the isolates into sequence types (STs) [8]. For each locus, a unique sequence is assigned an arbitrary and unique allele number. The designations for the seven loci are incorporated into an allelic profile (e.g. 1-1-1-1-1-1-1), and the ST (e.g. ST1) is assigned based on the allelic profile. STs can further be grouped into clonal complexes (CCs) based on the goeBURST algorithm [9]. The cutoff between CCs is usually defined as variation at one locus by most users [9]. The CCs are then named after the founder ST identified by the algorithm. For example, the founder ST of CC156 is ST156.

MLST is portable and designed to accommodate the conflicting signals of vertical and horizontal genetic transfer to infer evolutionary relationships [10]. Additionally, it is straightforward to compute and does not require whole-genome sequencing. Over the years, limitations of the MLST scheme have been observed as an increasing number of pneumococcal genomes become available. First, it is known that the absence or disruption of genes within the MLST scheme can prevent ST assignment, and preliminary data suggest that this is true for *gki* and *xpt* [11]. Second, a high recombination frequency of *gki*, *gdh*, *recP*, *spi* and *ddl* can obscure vertical genetic transfer, resulting in over-clustering due to convergent MLST profiles in phylogenetically disparate isolates [12,13]. For example, *ddl* has been observed in linkage with pbp, which is a recombination hotspot [14]. In addition, our previous study has identified that larger collections of genomes are more vulnerable to spurious connections when assigning CCs [3]. Similar observations have also been reported in other bacterial species [15]. Third, CC assignments can vary between collections, making the nomenclature inconsistent between studies. Finally, MLST has limited resolution capabilities – when applied to closely related isolates belonging to the same ST, the limited number of loci involved can lead to under-clustering, as it is unable to identify fine-grained evolutionary events that distinguish strains within a recent epidemic [16,17].

As more genomes became available, another gene-by-gene approach, core genome MLST (cgMLST), was devised to expand the number of genes included in the MLST analysis. Here, the analysis of the gene content of a selected subset of genomes is used to determine a set of genes that reflect the core genome. Clustering into groups is then based on the allelic profile of these core genes [18], often upwards of 1000 compared with the seven housekeeping genes in an MLST scheme, therefore increasing the resolution significantly and making better use of the bacterial genome compared with seven-allele MLST. The allelic profiles of the core genes are most commonly used for clustering, but they can also be used to construct phylogenetic trees using the SplitsTree4 package [19] or hierarchical clustering (HierCC) [20]. However, these phylogenies do not account for the nucleotide diversity within each locus as the allelic profile from the cgMLST scheme does not contain this information [18]. Although publicly available core genome schemes exist for many bacteria, cgMLST is not widely used in *S. pneumoniae* [18,20], which could be in part due to how laborious defining a core genome schema is and the difficulties in having it universally adopted. Using keywords ‘*Streptococcus pneumoniae* cgMLST’, only three publications were found (searched conducted on 10 April 2024) [21,23].

There is also whole genome MLST (wgMLST), which applies the principles of MLST to every open reading frame within a genome, therefore capturing the variation outside of the core genome. This was not specifically investigated in this work as various studies in bacteria such as *Campylobacter jejuni* [24], *Campylobacter coli* [24], *Pseudomonas aeruginosa* [25], *Clostridioides difficile* [26], *Salmonella enterica* [27,28], and *Listeria monocytogenes* [29] have shown clusters assigned by wgMLST to not be significantly different or superior to cgMLST assignments. wgMLST also poses other challenges; for example, accessory genes can be more difficult to align than core genes [30]. It is unclear if a wgMLST scheme should be continually expanded as novel accessory genes are identified [30], and there can be issues resolving paralogous and orthologous genes [31].

Recently, a related typing system – life identification numbers (LIN) – has been proposed, which makes use of cgMLST to provide each *S. pneumoniae* genome with a barcode based on the allelic similarity between it and other genomes in the dataset [18]. In this study, the final core genome schema was defined as 1222 genes [18]. For each genome, a species, super lineage, lineage, sublineage, clonal group and clonal subgroup are assigned based on the percentage of core gene allelic mismatches; for isolates in different lineages, this was defined as a mismatch of 540 genes (44.2% of the core genome schema) [18]. LIN barcoding has been used to study *Klebsiella*, which similarly to *S. pneumoniae* has high genomic diversity [32]. One benefit of the barcoding system is that each lineage has defined clusters within it, providing a high level of resolution beyond the lineage level. Additionally, it automatically keeps names from legacy schemes [32]. However, as it is based on cgMLST, it still does not account for nucleotide diversity within each locus and requires the creation of a scheme of core genes [18]. Different core gene sets and different cut-offs for allelic similarity may lead to different clustering results and thus inconsistent nomenclature.

An alternative reference-free approach based on k-mer similarity, as opposed to allelic profiles, has been proposed. Here, short nucleotide sequences of length k, known as k-mers, are used to measure core and accessory genome similarity between pairs of genomes and then cluster the genomes into lineages based on these distances. A reference database of genomes is used to ensure that nomenclature is consistent between studies. The software Population Partitioning Using Nucleotide K-mers (PopPUNK) [30] has been used to create genomic definitions of pneumococcal lineage (or Global Pneumococcal Sequence Clusters (GPSC)) on a global collection of ~20 000 genomes [3]. This approach grouped isolates that had shared evolutionary history into a GPSC based on variations across the entire genome in a scalable fashion with standard nomenclature. Generally, GPSCs had high concordance with ST/CC, and GPSC assignments often encompassed related CCs [3,33].

With the growing number of pneumococcal genomes available in the Global Pneumococcal Sequencing (GPS) project, discrepancies between GPSC and ST/CC have increasingly been observed. In this study, we compared the clustering results from the gene-by-gene-based approaches (MLST, cgMLST and LIN barcoding) and a k-mer-based approach (PopPUNK/GPSC). We then conducted an in-depth investigation into the discrepancies using phylogenetic and pan-genome analyses. Given that clustering should reflect the population structure, we would expect a successful clustering method to produce clusters that are monophyletic in the species-wide tree, showing high within-cluster similarity of genomic content.

## Methods

### Genome collection and *in silico* serotyping

In the GPS project, a global collection of 26 306 *S*. *pneumoniae* genomes was sequenced from 57 countries, 1989–2020 [34]. These published genomes passed quality control and were assembled as previously described for the global pneumococcal dataset [3,6]. Briefly, the sequence reads had a base quality >20, *S. pneumoniae* was the predominant species in Kraken with >60% species-specific reads, number of contigs assembled (<500), total assembled length (between 1.9 and 2.3 Mb), mapping coverage for *S. pneumoniae* reference (ATCC 700669, accession number FM211187 [35]) >60 %, overall coverage depth >20× and the number of heterozygous SNP sites <220. *In silico* serotyping was carried out using SeroBA with default parameters [36].

### Assignment of ST and CC

The pneumococcal genomes in this study were assigned STs using MLSTcheck version 2.1.17 [10,37]. The allelic profile of seven housekeeping genes (*aro*E, *gdh, gki*, *rec*P, *spi*, *xpt* and *ddl*) were input for clustering using the goeBURST algorithm based on a cutoff of single locus variants. The goeBURST algorithm is implemented in Phyloviz v2.0 [38]. The CC was named as per the ST identified as the primary founder by goeBURST.

### GPSC

For each genome in this study, GPSCs were assigned using PopPUNK v2.6.0 [30] with the reference database GPS_v6. The reference database contains 42 163 pneumococcal genomes and 1 112 GPSCs.

### cgMLST and HierCC

The genome assemblies were used to create cgMLST using chewBBACA v3.2.0 [39]. A subset of 100 randomly chosen genomes was first used to create the schema, and then the remaining 26 206 genome assemblies were used to detect the presence of alleles defined in the schema [39]. Together, the cgMLST profile for the entire collection was created, which was composed of 1 248 genes. The cgMLST profiles were then used to perform further clustering using HierCC v1.27 [40] with an allelic distance of 620, as this provided the highest silhouette score with HCCeval [40].

### LIN barcodes and lineages

LIN barcodes were accessed on 2 February 2023 [18] and incorporated into the dataset via ERR numbers. 17 322 genomes in our dataset were found in the Pneumococcal Genome Library (PGL) and so could be assigned LIN barcodes. 765 genomes in the LIN database did not have ERR numbers. Samples within our dataset that did not have a LIN barcode were not used for LIN-related analysis, but our full dataset of 26 306 genomes was used for CC, HierCC and GPSC analysis.

To compare lineage assignments between the methods, LIN barcodes were truncated to only include the first three numbers, which is the defined lineage level [18]. The lineage cut-off is defined by the authors of the PGL to be a mismatch of 540 genes in their cgMLST scheme [18].

### Single-cluster phylogenetic and pan-genome analyses

For the selected sets of genomes within a single cluster (one GPSC, one CC or one HierCC), we used Panaroo v1.3.3 [41] to identify core and accessory genome content. In tandem, Gubbins v3.2.1 [42,43] was used to build a recombination-free phylogeny for every single cluster of genomes analysed (e.g. a single GPSC or a single CC). The reference sequence used for each cluster was that of the dominant GPSC. This tree along with core and accessory genome content was visualized in Phandango [44].

### Species-wide phylogenetic analyses

Pseudo-genomes were created using samtools mpileup after mapping the genomes to the *S. pneumoniae* reference genome (ATCC 700669, accession number FM211187). SNP sites were identified from the pseudo-genomes using snp-sites [45]. FastTree v2.1.10 [46] was used to construct a maximum likelihood phylogenetic tree using the SNP sites across all 26 306 genomes. FastTree [46] used a generalized time-reversible model and gamma rate substitution. The species-wide tree and associated data can be visualized on Microreact (https://microreact.org/project/pneumolineageanalysis).

### Statistical analysis

The adjusted mutual information (AMI) score measures the agreement between clustering methods after adjusting for the agreement expected between random clustering. The score ranges between −1 and 1, whereby −1 indicates complete disagreement, 0 that the clustering is no better than random and 1 that there is complete agreement between clustering methods. The function AMI from the R package aricode was used [47].

To compare between the methods, the mean (μ) and standard deviation (σ) of the number of clusters produced by method A within a single cluster of method B were calculated in R.

## Results

### Genome collection summary

The global collection of pneumococcal genomes (*n*=26 306) was derived from isolates causing invasive disease (*n*=12 063), non-invasive disease (*n*=630) and asymptomatic colonization (*n*=7 948) from 57 countries, 1989–2020 (Data S1). Of 26 306 pneumococcal genomes, 81 in silico serotypes were identified. A subset of 17 361 genomes within our database had associated LIN barcodes from the Pneumococcal Genome Library, and so these were incorporated.

### Overview of clustering methods

**Fig. 1.**
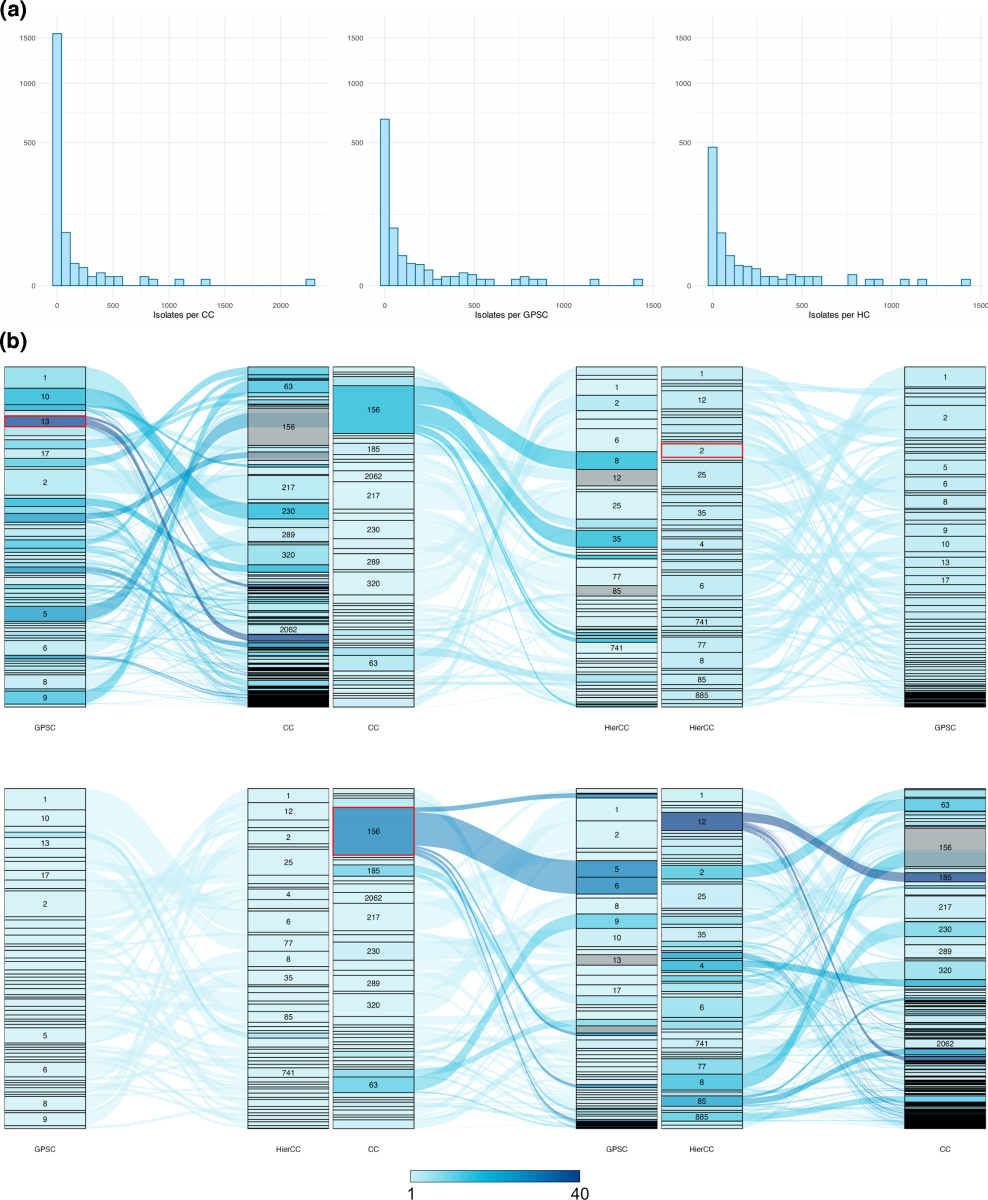
Number of genomes in one cluster for each method. (**a**) Many CCs contain just one or very few genomes, compared with GPSC and HierCC clusters. (**b**) Sankey plots to visualize the difference between methods. Each left bar represents clusters with over 100 genomes determined by one method, shown as being distributed into clusters defined by a second method, represented by the bar on the right. The colour shows the number of clusters produced by method two for a single cluster from method one; the darker the blue, the more clusters it has been split into. Clusters analysed in further depth in the results are highlighted in red.

For 26 306 pneumococcal genomes, 4 766 STs were identified and grouped into 1 656 CCs. Of these, 801 (48%) CCs were only composed of a single ST (i.e. were singletons). 25 812 cgMLSTs were identified in the same set of pneumococcal genomes, which were clustered into 606 HierCCs in which 146 (24%) were singletons. Using PopPUNK, 830 GPSCs were identified of which 272 (30%) were singletons (Table 1). Given the differences in the number of total clusters, the mean number of sequences within each cluster varied accordingly; methods that led to fewer total clusters ended up with more genomes per cluster (Table 1, Fig. 1a). This decreases the resolution of the method and potentially leads to difficulties in tracking small-scale outbreaks of disease. On the other hand, as the number of total clusters increases, so does the proportion of singletons, and so the benefits that come from clustering are reduced.

**Table 1.** Statistical analysis of the results of the three different clustering methods AMI scores are shown to report the concordance between methods. Mean (*μ*) and standard deviations (*σ*) are shown for the number of genomes per cluster for each method. Additionally, we show the mean and standard deviation for the number of one cluster type within a single cluster of a different type (e.g. the mean number of GPSCs within a single CC; the mean number of HierCCs within a single LIN lineage).

		CC	HierCC	GPSC	LIN Lineage*
Total clusters		1656	606	830	609
No. of singletons (%)		801 (48%)	146 (24%)	272 (30%)	256 (42%)
Max genomes in a single lineage		2265	1417	1417	957
Min genomes in a single lineage		1	1	1	1
AMI vs. CC			0.835	0.869	0.875
AMI vs. HierCC		0.835		0.946	0.950
AMI vs. GPSC		0.869	0.946		0.979
AMI vs. LIN		0.875	0.950	0.979	
Genomes per lineage	*μ*	15.9	43.4	31.7	28.4
	*σ*	86.65	135.84	109.19	82.6
A single CC	*μ*		1.04	1.07	1.07
	*σ*		0.33	0.70	0.57
A single HierCC	*μ*	2.83		1.38	1.29
	*σ*	4.77		1.43	0.95
A single GPSC	*μ*	2.13	1.01		1.09
	*σ*	2.95	0.08		0.42
A single LIN lineage	*μ*	2.04	1.00	1.07	
	*σ*	2.59	0.04	0.36	

*For this analysis, only the 17 322 genomes from our dataset with associated LIN lineages were included.

AMI scores were used to compare the level of agreement between different clustering methods. The AMI score between CC and GPSC assignments was 0.869, showing high concordance between the two methods; 97% (1608/1656) of CCs were composed of genomes belonging to a single GPSC. After removing singleton CCs (*n*=801), this changed to 94% (807/855). The AMI score between HierCC and GPSC clustering was 0.946, showing that there is greater concordance between HierCC and GPSC assignments than CC and GPSC assignments. In the subset of the data which had assigned LIN barcodes, the AMI score between HierCC and LIN lineages was 0.950, showing almost complete concordance but still a degree of variability likely introduced through the selection of the core genome schema and different definitions of lineage. Interestingly, this shows that despite both using core genome schemes, HierCC and LIN lineages are as similar to each other as HierCC and GPSC assignments are to each other. Overall, CC assignments were the most different from the other methods (Fig. 1b).

None of the methods produced identical results to each other. To investigate these differences further, we selected examples for further analysis where a single cluster produced using one method was split into multiple different clusters by another method. For example, there were 48 (3%) CCs containing genomes belonging to multiple (>1) GPSCs and 285 (35%) GPSCs with genomes belonging to >1 CCs.

### CCs containing multiple GPSCs

To learn more about CCs containing multiple GPSCs, we investigated all the CCs containing at least 500 genomes. There were eight CCs that met these criteria (CC156, *n*=2 265; CC217, *n*=1 299; CC320, *n*=1 085; CC230, *n*=864; CC289, *n*=754; CC63, *n*=742; CC185, *n*=537; CC2062, *n*=520). Of these, four consisted of a single GPSC (CC217, CC320, CC289, CC2062) and so were not investigated further. However, the others all contained multiple different GPSCs (CC156, *n*=23; CC63, *n*=10; CC185, *n*=9; CC230, *n*=2). As CC156 contained the greatest number, we investigate this example in further detail here. Figures for the other CCs that contained multiple GPSCs can be found in the supplementary (Figs S1–S3).

**Fig. 2.**
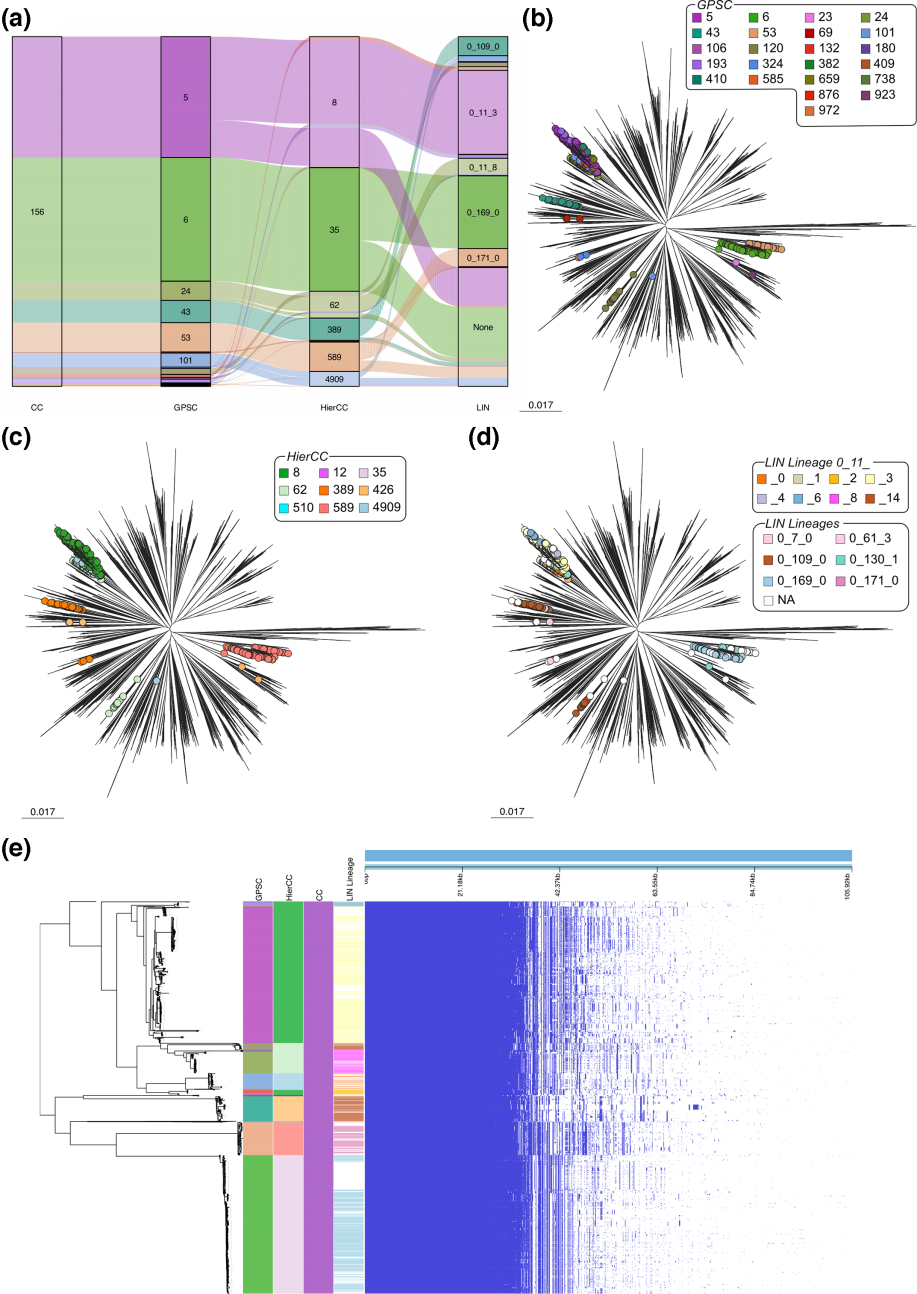
Analysis of genomes assigned to CC156. (**a**) Within CC156, there were multiple GPSCs, HierCCs and LIN lineages. (**b**) In a species-wide tree, the genomes within CC156 were spread across the whole phylogeny. GPSCs (b), HierCCs (c) and LIN lineages (**d**) were better able to cluster according to the phylogeny. (**e**) Pan-genome analysis of CC156 with a recombination-free tree. There are clear differences in genomic content between genomes, broadly correlating with GPSC, HierCC and LIN lineage assignments.

CC156 contained 23 different GPSCs, with GPSC5 and GPSC6 being the dominant GPSCs. This corresponded to 9 HierCCs and 14 LIN lineages (Fig. 2a). Within these LIN lineages, there were seven different superlineages, suggesting large and substantial differences between the genomes (Fig. 2a). The genomes within CC156 form a polyphyletic group and do not cluster in the species-wide phylogeny, being spread across multiple distant branches of the tree, which supports the assignment to different superlineages and lineages. When coloured by GPSC, it is possible to see that individual clades within the tree have been assigned to different GPSCs (Fig. 2b), and a similar pattern is true for HierCC and LIN lineages (Fig. 2c, d).

Pan-genome analysis of CC156 shows the concordance between GPSC, HierCC and LIN lineage assignments of the genomes within CC156 (Fig. 2e). Visual differences in the gene content can be observed between the different major HierCCs, GPSCs and LIN lineages, supporting even further the subdivision of CC156 into multiple groupings. Interestingly, we also found that of CCs analysed, CC156 had the smallest core genome size, defined as genes which are present in 99–100% of genomes within the cluster (CC63, 1 618 core genes; CC156, 1 569 core genes; CC185, 1 628 core genes; CC230, 1 648 core genes). However, for the assignments of minor groupings, it is difficult to conclusively determine if this subdivision is biologically relevant and valid.

Together, this shows that the few genes used to define a CC are likely to group together distantly related genomes, creating difficulties when trying to track epidemics and when discussing lineages. In this case, CC156 contains genomes within ST156, which has been identified as a genotype of high concern due to its rapid spread and high resistance to penicillin and cotrimoxazole [35].

### GPSCs containing multiple CCs

We investigated the GPSCs containing at least 500 genomes to identify GPSCs containing multiple CCs. There were nine GPSCs that contained over 500 genomes, and each of these contained multiple CCs (Table 2). As GPSC13 contained the most CCs, we investigate it in further depth here. However, figures for the other GPSCs containing multiple CCs can be found in the supplementary (Figs S4–S11).

**Table 2.** GPSCs with over 500 genomes and the number of CCs contained within each

GPSC	No. of genomes	No. of CCs	Dominant CC (no. of genomes)
1	1 166	9	CC320 (1,085)
2	1 417	5	CC217 (1,299)
5	835	24	CC156 (783)
6	803	4	CC156 (800)
8	765	3	C289 (754)
9	713	16	CC63 (683)
10	897	19	CC230 (862)
13	592	35	CC2285 (347)
17	524	4	CC2062 (520)

**Fig. 3.**
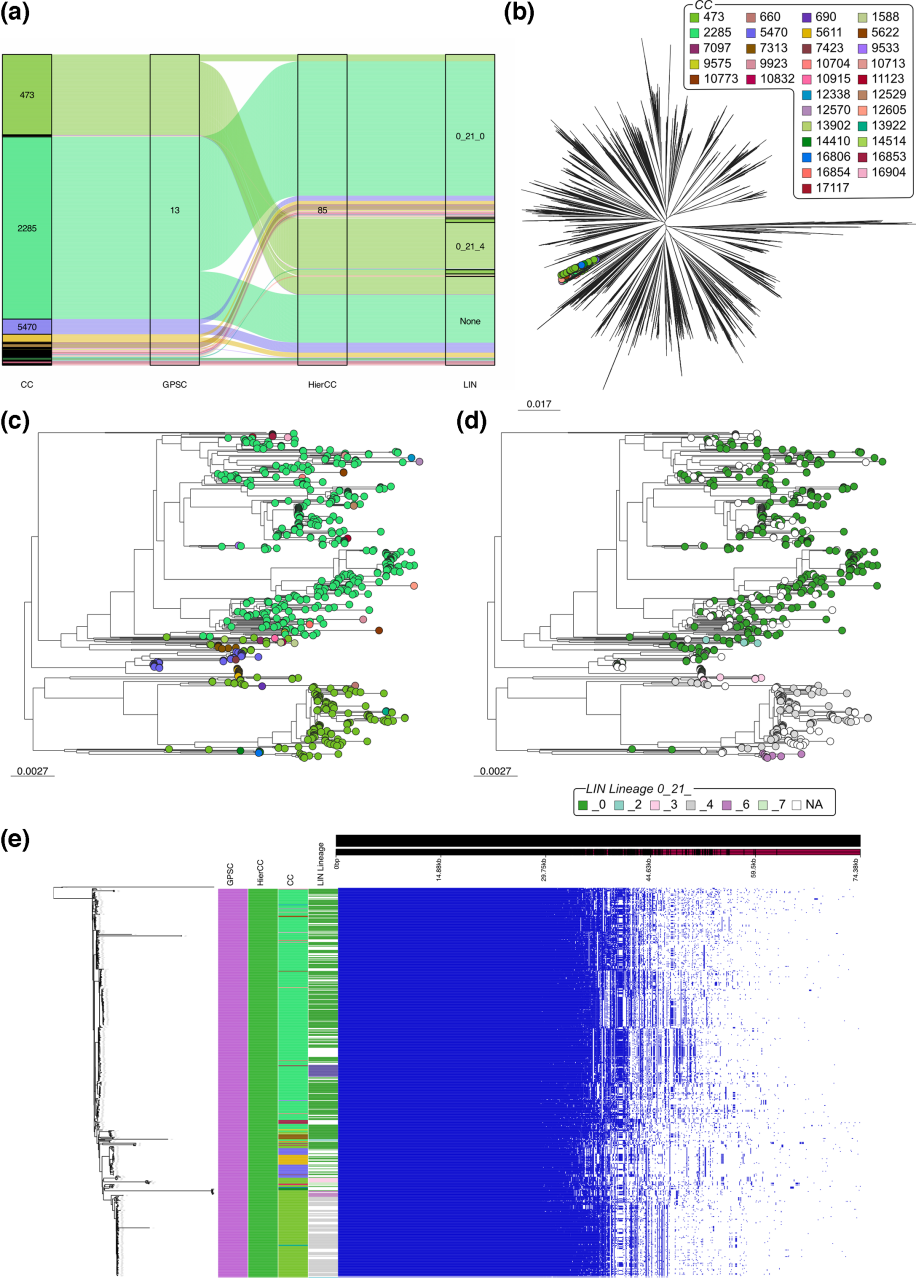
Analysis of genomes assigned to GPSC13. (**a**) Within the single GPSC and HierCC, there were multiple CCs and LIN lineages. (**b**) In a species-wide tree, the genomes within GPSC13 clustered together. There was limited clustering at the CC (b, **c**) level and LIN lineage (**d**) level. (**e**) Pan-genome analysis with a recombination-free tree of GPSC13 genomes showed high similarity between the genomes, not supporting the high number of CC assignments.

GPSC13 is equivalent to HierCC 85; however, it is split into 35 CCs (Fig. 3a). As this is the greatest number of CCs seen within a single GPSC in this dataset, we performed a thorough analysis of it. The genomes within GPSC13 formed a cluster within the species-wise phylogeny; however, the clade was not monophyletic (Fig. 3b). The subtree of GPSC13 showed that the two dominant CCs, CC473 and CC2285, formed distinct groupings (Fig. 3c). However, there were many minor CCs scattered within two, with 68.6% of the CCs only containing a single genome. 88.6% of the CCs within GPSC13 were singletons, containing a single ST that was not found elsewhere in the database. GPSC13 contained six different LIN lineages, all of which were within the 0_21 super lineage (Fig. 3d), reflecting their close relatedness. These lineages sat in clusters in the subtree.

The same phylogenetic clustering was seen in the recombination-free GPSC13 phylogeny (Fig. 3e). Pan-genome analysis of the genomes within GPSC13 showed them to be similar to each other, with a core genome (>95%) size of 1 743 genes. The different CCs and LIN lineages do not correspond to any clear visual changes in genome content from the pangenome analysis (Fig. 3e), suggesting that the further subdivision is not reflective of significant changes in the genome.

We performed the same analysis on the other GPSCs containing multiple CCs, and the figures can be found in the supplementary. Interestingly, we continued to see the same pattern of GPSCs and HierCCs being concordant with each other, whilst there are multiple CCs present. Often, the genomes are dominated by one CC with multiple minor CCs containing very few genomes. This suggests that CC assignments are likely to be noisy.

### HierCCs containing multiple GPSCs

**Fig. 4.**
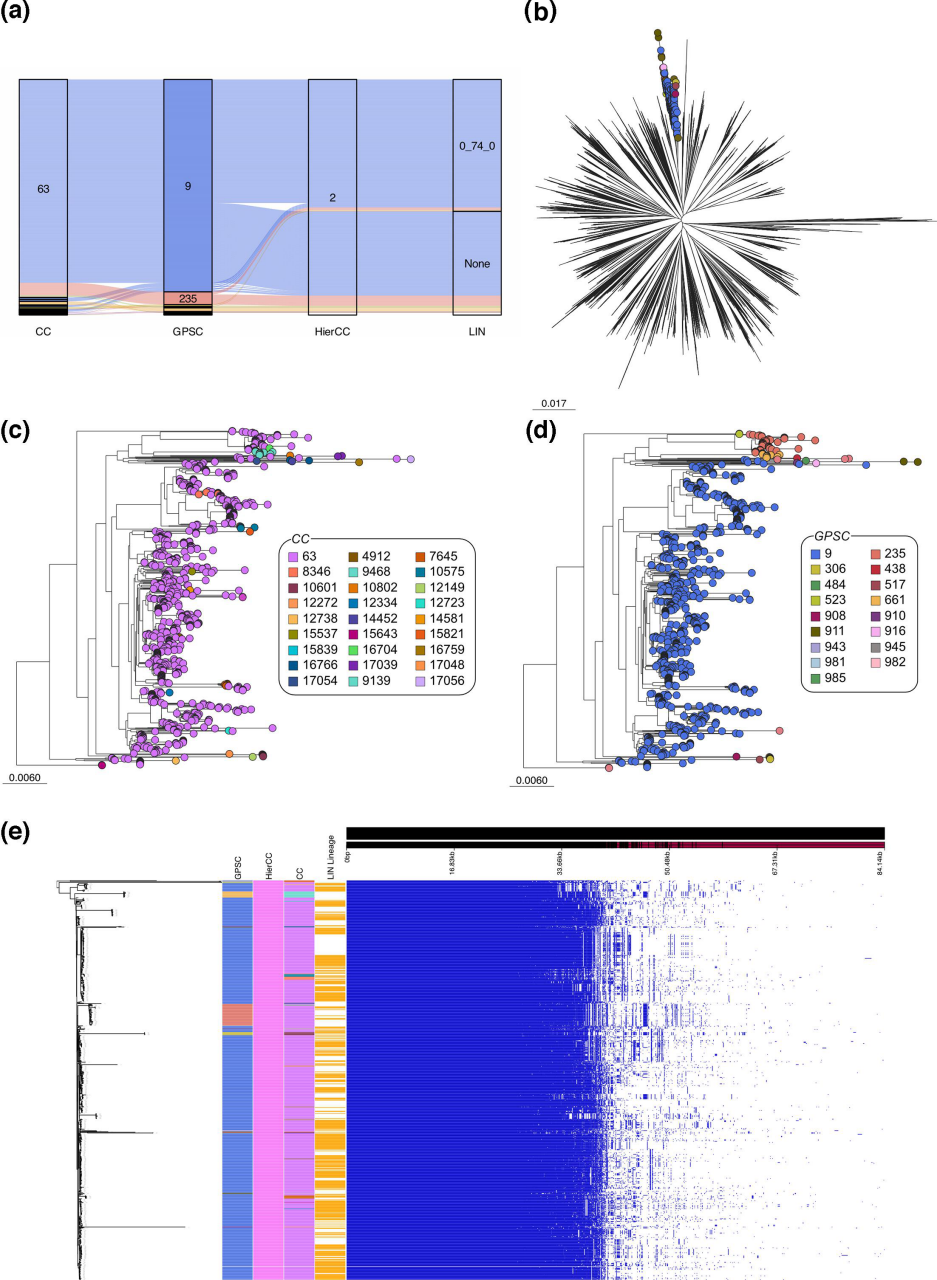
Analysis of genomes assigned to HierCC 2. (**a**) Within the one HierCC, there were multiple GPSCs and CCs. (**b**) In a species-wide tree, the genomes within HierCC 2 were in the same branch. (**c**) CC63 is the dominant CC, with many minor CCs spread throughout. (**d**) The two main GPSCs, GPSC9 and GPSC235, formed two clusters, with other minor GPSCs. (**e**) Pan-genome analysis with a recombination-free tree of HierCC two genomes showed high similarity between the genomes. Differences in the accessory genome rationalize the existence of GPSC235 as a separate cluster.

83.3% (505/606) of HierCCs contained a single GPSC, and therefore 16.6% (101/606) HierCCs contained multiple GPSCs. We identified the five HierCCs that contained the most GPSCs for further analysis (HierCC 2, *n*=791; HierCC 12, *n*=1 063; HierCC 8, *n*=933; HierCC 390, *n*=257; HierCC 885, *n*=515). The greatest number of GPSCs was seen in HierCC 2, which contained 17 GPSCs and 27 CCs (Fig. 4a), and so we analysed this further.

The genomes in HierCC 2 only belonged to a single LIN lineage, 0_74_0, showing the similarity between the two cgMLST methods despite the schema differences. All the genomes within HierCC 2 are positioned within a single branch of the species-wide phylogeny (Fig. 4b). Colouring the subtree by CC showed that the vast majority of genomes were assigned to CC63, with many other minor CCs spread throughout (Fig. 4c). 66.7% (18/27) of these were represented by only a single genome. These single genomes, assigned to different CCs, despite clustering with other CC63 isolates throughout the phylogeny, indicating that relying solely on the CC designation may lead to clinical expansions or outbreaks being missed. Colouring the subtree by GPSC showed that the majority of samples belonged to GPSC9 or GPSC235, with each of these forming distinct clusters (Fig. 4d). Genomes belonging to other GPSCs can be seen, often clustering together.

The recombination-free phylogeny of HierCC 2 genomes also showed clustering by GPSCs (Fig. 4e). Pan-genome analysis supported the assignment of the genomes within GPSC235 into their own cluster, as shown by clear differences in the accessory genome compared with other genomes (Fig. 4e). These genes include some from transposons, recombinases and hypothetical proteins amongst others (Table S1). This difference was not identified by the HierCC, CC or LIN lineage assignments, reflecting whether or not they account for the accessory genome.

We performed the same analysis on the other HierCCs that contain multiple GPSCs, and the figures can be found in the supplementary (Figs S12–S15).

### GPSCs containing multiple HierCCs

**Fig. 5.**
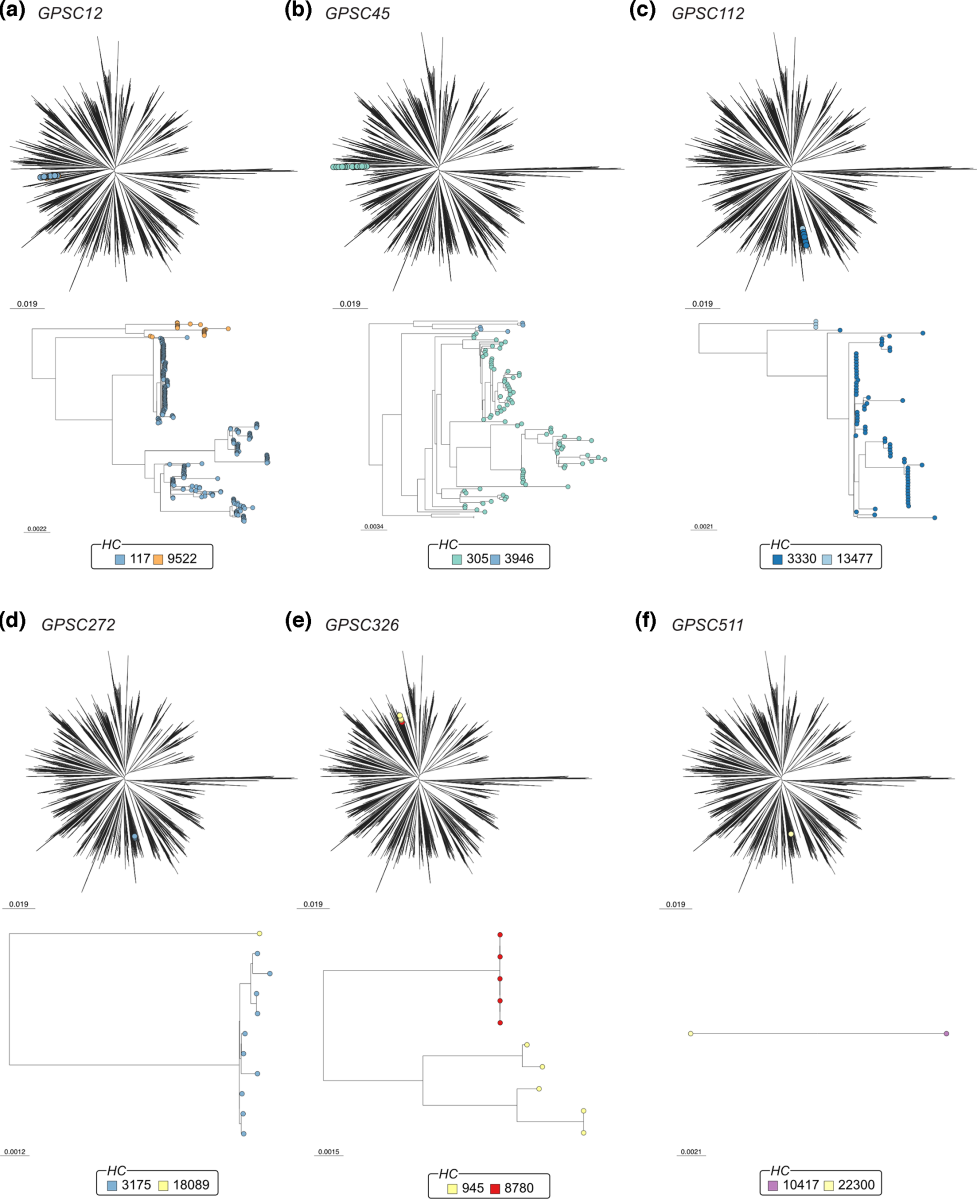
Analysis of the six GPSCs containing two HierCCs. (**a**) GPSC12, (**b**) GPSC45, (**c**) GPSC112, (**d**) GPSC272, (**e**) GPSC326 and (**f**) GPSC511 each contain two HierCCs. The genomes within each GPSC cluster in species-wide phylogenies, and in the subtrees, the separation by HierCC was evident.

There were very few GPSCs containing multiple HierCCs; the mean number of HierCCs per GPSC was 1.01. Only six GPSCs contained two HierCCs (GPSC12, GPSC45, GPSC112, GPSC272, GPSC326 and GPSC511). Each of these GPSCs occupies the same branch of the phylogeny, indicating high relatedness (Fig. 5). Individual subtrees showed clustering of the two HierCCs into different branches, suggesting that HierCCs provide slightly higher resolution clustering than GPSCs (Fig. 5). Given the few genomes within each GPSC, pan-genome analysis was not performed, as reasonable conclusions cannot be drawn from such few samples about the validity of the clustering.

### HierCCs and LIN lineage discordance

Both HierCCs and LIN lineages are based around cgMLST. However, they have different core gene schema (1 222 genes in the LIN lineage core gene schema [18] and 1 248 in our core gene schema) and different criteria for defining separate lineages; for HierCCs, clustering is defined as the allelic distance which provides the maximal silhouette score for the dataset [40], whereas LIN lineages are based on the percentage allelic difference between the genomes. In the subset of data that was assigned a LIN lineage from the PGL (*n*=17 322) [18], there were 474 different HierCCs and 609 different LIN Lineages. There was a mean of 1.00 HierCCs per LIN lineage and 1.29 LIN lineages per HierCC, showing that LIN lineages are more subdivided than HierCC assignments. As both of these methods are cgMLST-based, we decided to investigate the discrepancies between HierCCs and LIN lineages.

**Fig. 6.**
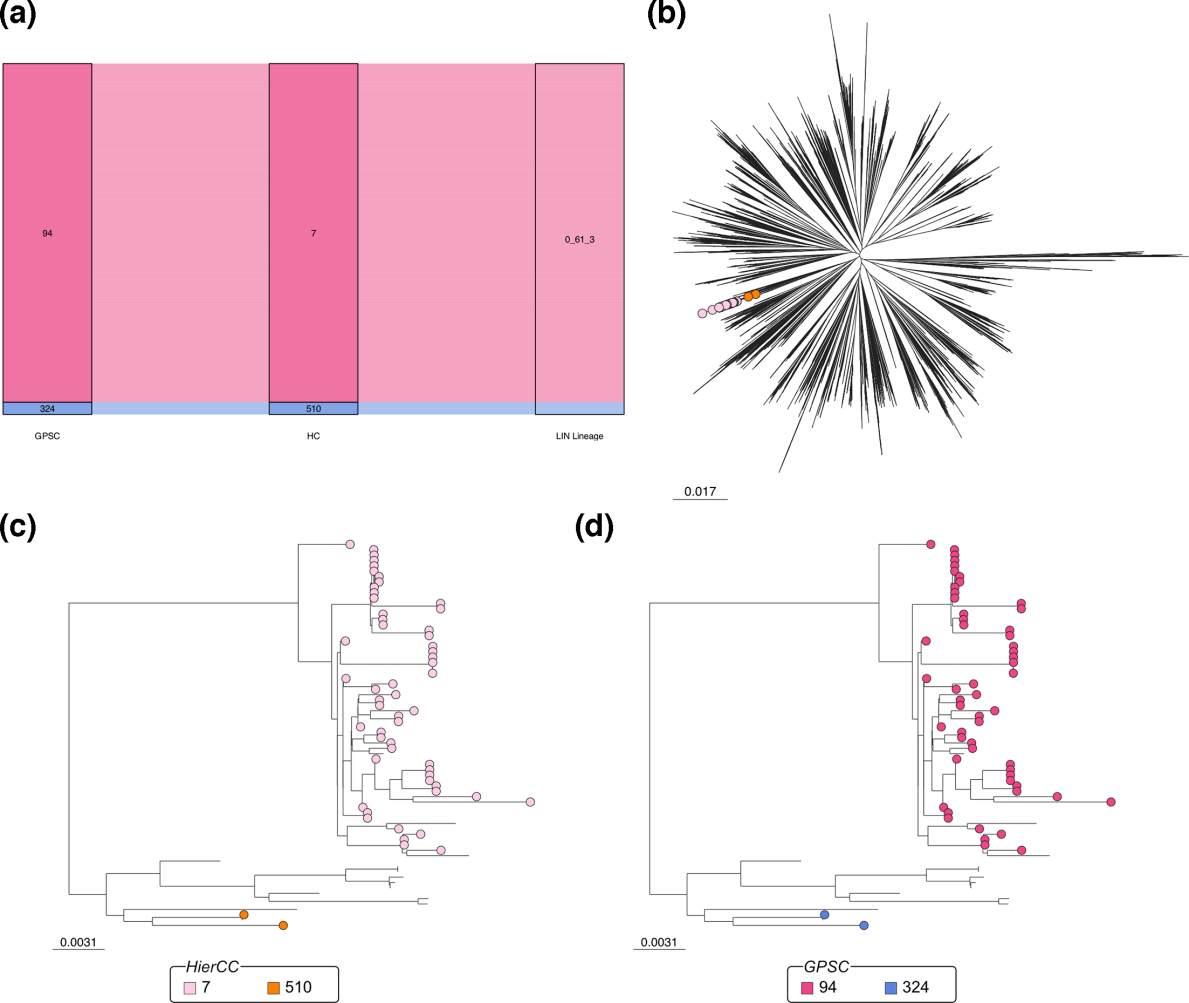
Analysis of genomes assigned to LIN lineage 0_61_3. (**a**) Both GPSC and HierCC assignments agreed that lineage 0_61_3 should be further divided into two clusters. (**b**) In a species-wide tree, the genomes were in the same branch. (**c**) The two HierCCs within 0_61_3 split into two different branches of the subtree. (**d**) The same is true for the GPSCs.

Only one LIN lineage contained multiple HierCCs; this was 0_61_3 (*n*=57), which contained members of HierCCs 7 (*n*=55) and 510 (*n*=2) (Fig. 6a). All of the genomes clustered together in the species-wide phylogeny; however, the cluster is polyphyletic, with closely related genomes being assigned to lineage 0_61_4 (Fig. 6b). However, HierCC 7 and HierCC 510 were both part of distinct clades in the subtree (Fig. 6c). Notably, members of HierCC 510 had a visibly shorter branch length than those in HierCC 7, suggesting that they are distinct lineages and supporting the HierCC assignment into two clusters (Fig. 6c, d). Finally, the GPSC assignments (GPSC94, *n*=55; GPSC324, *n*=2) were entirely concordant with the HierCC assignments (Fig. 6d), supporting the division of these genomes into two clusters.

HierCC 390 and HierCC 885 both contained ten LIN lineages, but as they contained multiple CCs and GPSCs, they are discussed earlier in the paper, and the figures can be found in the supplementary (Figs S14 and S15).

### CC assignment discordance

As we used data from the PGL to assign LIN barcodes where possible, it is possible to compare the differences between CC assignments in our dataset and CC assignments from the PGL dataset. There was a 14.5% mismatch between CCs assigned in our dataset and the PGL dataset, showing that the CC nomenclature is contingent on the sample collection.

## Discussion

Accurate clustering of pathogen genomes is a key part of defining the population structure, as it is likely that closely related isolates may have similar clinical properties such as the disease potential [3], transmissibility [4] and antimicrobial resistance [3]. It can also be useful for identifying transmission of *S. pneumoniae* within a population during an epidemic. Additionally, consistent nomenclature is important when communicating results in the global context. Clustering needs to be high resolution such that differences between groups can be distinguished, but not so fine-grained that each cluster only has one or two members, and so the benefits of clustering are lost.

In this study, we used a large and highly diverse global collection of high-quality *S. pneumoniae* genomes to compare the clustering performance of k-mer-based PopPUNK (GPSC assignments) in comparison to MLST-based CC assignments and cgMLST-based HierCC and LIN lineage assignments. AMI scores between the different clustering methods show that the results are broadly consistent with each other. However, where they do differ, CCs tend to give the poorest reflection of the population structure based on the species-wide phylogeny, with genomes from a single CC often being phylogenetically distant from each other. Additionally, differences in the collection of genomes used to assign CCs may lead to differences in cluster names. Such differences could present a barrier to effective genomic surveillance of *S. pneumoniae*. The two cgMLST methods, HierCC and LIN lineages, as well as k-mer-based PopPUNK, are highly concordant, and their clustering assignments are validated by phylogenetic analysis and pan-genome. Therefore, each of these three methods offer a valid approach to defining the population structure of *S. pneumoniae* genomes.

Each of these methods has its own advantages and disadvantages (Table 3). All cgMLST methods require the definition of a core genome schema, which can differ from study to study depending on the cut-off of prevalence of the gene within the population used to establish a gene as core, and the collection of genomes used to determine the scheme. This can lead to different core genome profiles being used between studies, and potentially different assignments in clustering, as shown by discrepancies between LIN lineages and HierCC assignments. When considering the global spread of *S. pneumoniae*, consistency between studies is key for the accurate interpretation of results and communication between researchers. Additionally, the definition of core genomes can take a significant amount of time. In contrast to this, PopPUNK does not require a core genome schema, leading to greater consistency between studies and faster results, which can be important when tracing an outbreak and describing epidemiology. PopPUNK is the only approach which makes use of the whole genome, including intergenic regions and the accessory genome, instead of just the core genome. However, it still distinguishes between divergence in the core and accessory genomes. This information can be the difference between one cluster or two, as shown with GPSC9 and GPSC235 in HierCC 2.

**Table 3.** *S*ummary table of the advantages and disadvantages of different *S. pneumoniae* lineage assignment methods

Method	Advantages	Disadvantages
Seven-locus MLST (CC)	Quick and easy Common in the literature Does not require whole-genome sequencing	Low resolution due to only seven loci Known linkage between the seven loci Does not reflect the population structure well Requires the rerunning of all genomes to assign new members to a lineage Assignments vary based on the dataset
cgMLST (HierCC)	Uses more genes than seven-locus MLST Reflects the population structure well	Requires the definition of a core gene schema, which can differ between studies Nomenclature does not show relatedness Does not make use of the accessory genome Requires whole-genome sequencing Requires the rerunning of all genomes to assign new members to a lineage Assignments vary based on the dataset
cgMLST (LIN barcoding)	Unique barcode for each genome; includes information at the sublineage level Reflects the population structure well	Requires the definition of a core gene schema, which can differ between studies; does not make use of the accessory genome Requires whole genome sequencing No merging of clusters possible
K-mer (PopPUNK GPSC assignments)	Can add new genomes without rerunning the whole dataset No core schema definition required Merging is possible as new data are added, and the history of merges is retained in the nomenclature Makes use of the whole genome Reflect the population structure well	Requires whole genome sequencing

Another key difference between the methods is the ability for clusters to merge over time as new data are added, which is possible for both HierCCs and GPSCs. This can be of benefit, as new genomes support the grouping of the clusters as a better reflection of the population structure. However, this can lead to inconsistencies developing over time as GPSCs or HierCCs are absorbed into each other and previous literature is not updated. In contrast to this, the LIN barcoding system does not allow for merging,as each genome is provided with a unique barcode. Therefore, the nomenclature will be more stable long term. The disadvantage of this is that, if the initial sampling is poor or unbalanced, genomes may be assigned to different clusters initially, but if later data show that they belong to the same cluster, this cannot be changed. Additionally, although not a factor for *S. pneumoniae* due to the equidistant nature of strains, in other species, the barcode nature of LIN assignments allows users to easily identify the relatedness between two different LIN barcodes based on the nomenclature, whereas the nomenclature of HierCCs and GPSCs does not reflect the hierarchical clustering. For example, it is easy to tell that lineages 0_21_0 and 0_21_1 are closely related, but not how closely related CC63 and CC8346 are. However, at the time of writing this manuscript, it is not possible to assign LIN barcodes to data not already within the PGL database.

This work focused on comparing the methods for a single species only, and so cannot be generalized to other species without further detailed analysis. We used very stringent criteria to define clonal complexes (single-locus variants, only), and altering this could lead again to different clustering results. However, this again reflects the variability in methods. Additionally, further work investigating the validity of minor groupings, such as those with very few genomes, is required to determine the additional value of further subdivision where methods disagree. A final limitation is that wgMLST was not investigated in this study.

As there are now multiple methods available that use genomic data to provide higher resolution clustering than CC assignments, each with different advantages/disadvantages, it makes sense for the research community to transition to these methods over seven-locus MLST. However, to allow for easy comparison between studies and avoid making previous literature redundant, the reporting of multiple clustering names should be standardized within research. Additionally, resources to allow conversion between nomenclatures should be utilized, such as those on the GPS project website (https://www.pneumogen.net/gps/#/resources#gpsc-st-lookup-table) and the *Streptococcus pneumoniae* serotypes table (https://pneumococcalcapsules.github.io/serotypes/). This will allow for greater sense of collaboration within the field and improve open research practices.

## supplementary material

10.1099/mgen.0.001278 Uncited Fig. S1.

10.1099/mgen.0.001278 Uncited Table S1.

## Abbreviations

AMI: adjusted mutual information
CCs: clonal complexes
cgMLST: core genome MLST
GPS: Global Pneumococcal Sequencing
GPSC: Global Pneumococcal Sequence Clusters
HierCC: hierarchical clustering
LIN: life identification number
PGL: Pneumococcal Genome Library
PopPUNK: Population Partitioning Using Nucleotide K-mers
ST: sequence types
wgMLST: whole-genome MLST
