# Supplementary material for: Comparison of gene-by-gene and genome-wide short nucleotide sequence-based approaches to define the global population structure of Streptococcus pneumoniae
Source: Microb Genom. 2024 Aug 28;10(8):001278. doi: 10.1099/mgen.0.001278 (PMC11353345; doi:10.1099/mgen.0.001278)
Supplement: Uncited Fig. S1. [file mgen-10-01278-s001.pdf]

Supplementary Figures

Supplementary Figures *1*

Supplementary Figure 1 - Analysis of genomes assigned to CC63. 2

Supplementary Figure 2 - Analysis of genomes assigned to CC185. 4

Supplementary Figure 3 - Analysis of genomes assigned to CC230. 6

Supplementary Figure 4 - Analysis of genomes assigned to GPSC1 8

Supplementary Figure 5 - Analysis of genomes assigned to GPSC2 10

Supplementary Figure 6 - Analysis of genomes assigned to GPSC5 12

Supplementary Figure 7 - Analysis of genomes assigned to GPSC6 13

Supplementary Figure 8 - Analysis of genomes assigned to GPSC8 15

Supplementary Figure 9 - Analysis of genomes assigned to GPSC9 16

Supplementary Figure 10 - Analysis of genomes assigned to GPSC10. 18

Supplementary Figure 11 - Analysis of genomes assigned to GPSC17. 20

Supplementary Figure 12 - Analysis of genomes assigned to HierCC 8. 22

Supplementary Figure 13 - Analysis of genomes assigned to HierCC 12. 24

Supplementary Figure 14 - Analysis of genomes assigned to HierCC 390. 25

Supplementary Figure 15 - Analysis of genomes assigned to HierCC 885. 27

Supplementary Table 1 - A selection of genes present in GPSC235, but not in GPSC9, despite both sets of genomes being clustered into HierCC2. 30

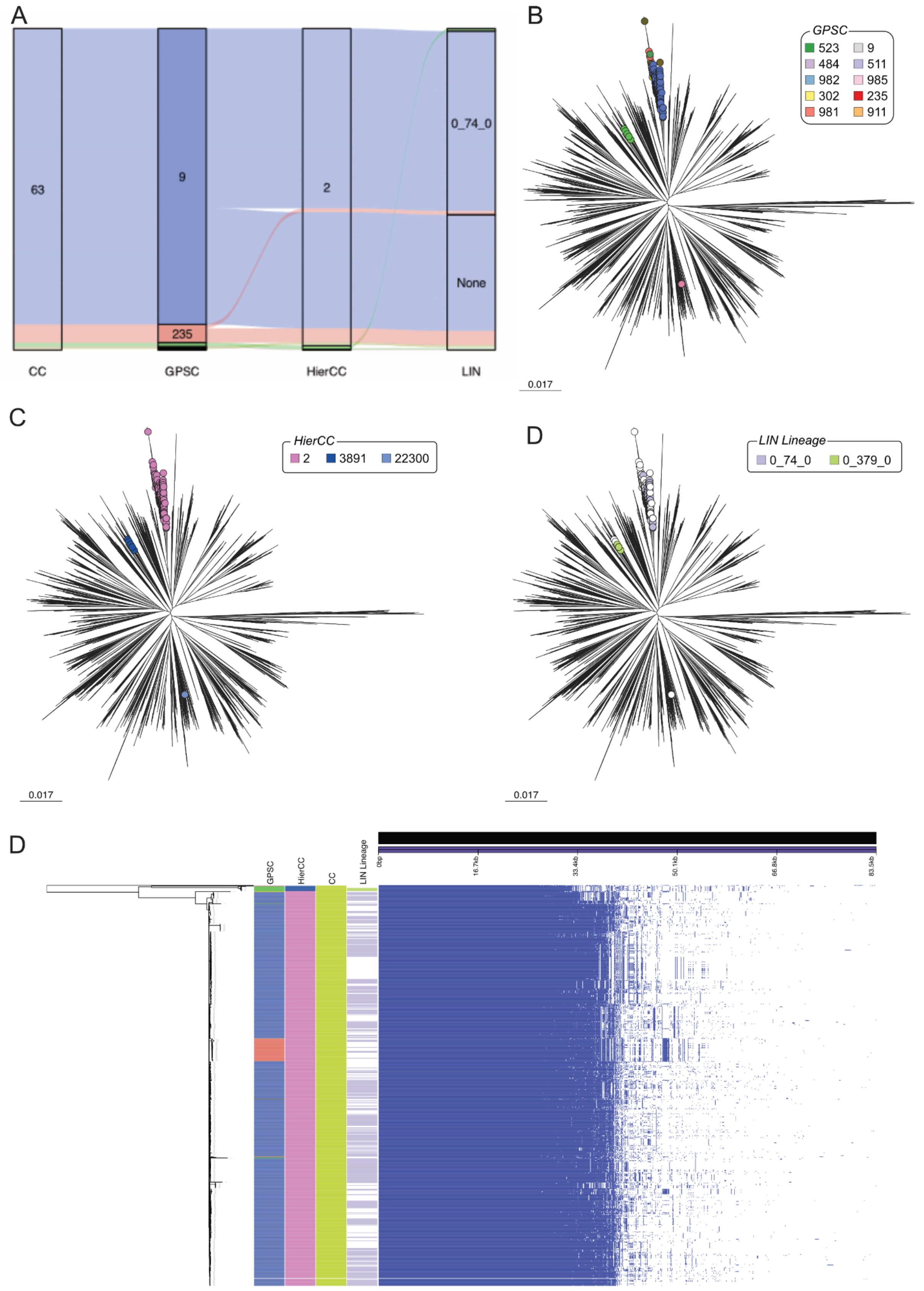

Supplementary Figure 1 - Analysis of genomes assigned to CC63. *(A) CC63 is split into multiple GPSCs, HierCCs, and LIN lineages. (B) In a species-wide phylogeny, the genomes clearly belong to distinct branches, reflecting the differences between the genomes and supporting their further subdivision. GPSCs are able to do*

this. (C) HierCC also clusters the genomes by tree location. (D) The genomes are in different LIN superlineages as well as lineages. (E) Pan-genome analysis supports the separation of CC63 into separate clusters.

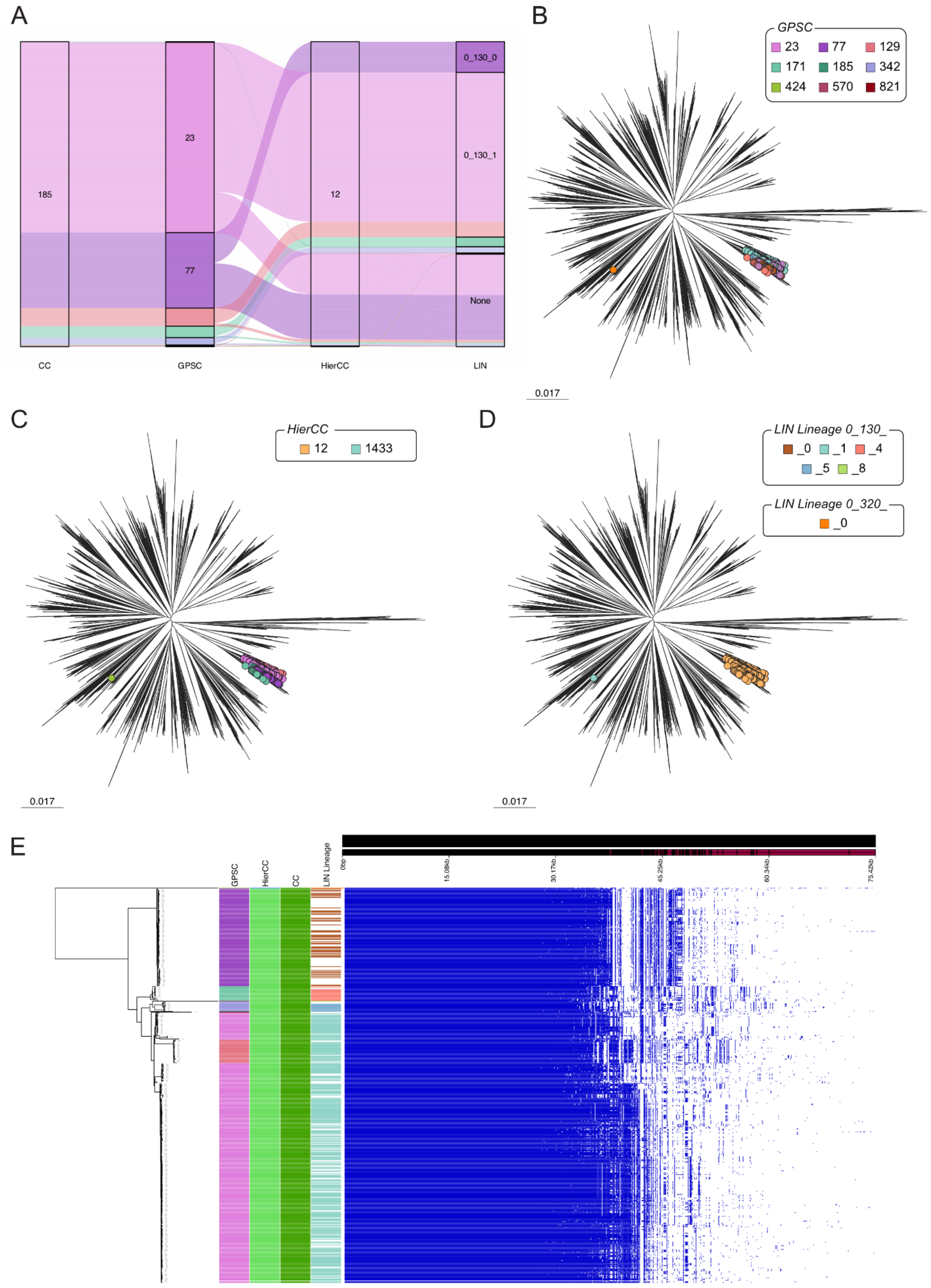

Supplementary Figure 2 - Analysis of genomes assigned to CC185. *(A) CC185 is split into multiple GPSCs and LIN lineages, but one HierCC. (B) In a species-wide phylogeny, the genomes clearly belong to distinct branches. GPSCs, (C) HierCC, and (D) LIN lineages are able to distinguish this. The genomes are in different LIN*

superlineages. (E) Pan-genome analysis supports the separation of CC185 into separate clusters, showing differences in the accessory genomes that have been lost in HierCC assignments, but recognised in LIN lineages and GPSCs.

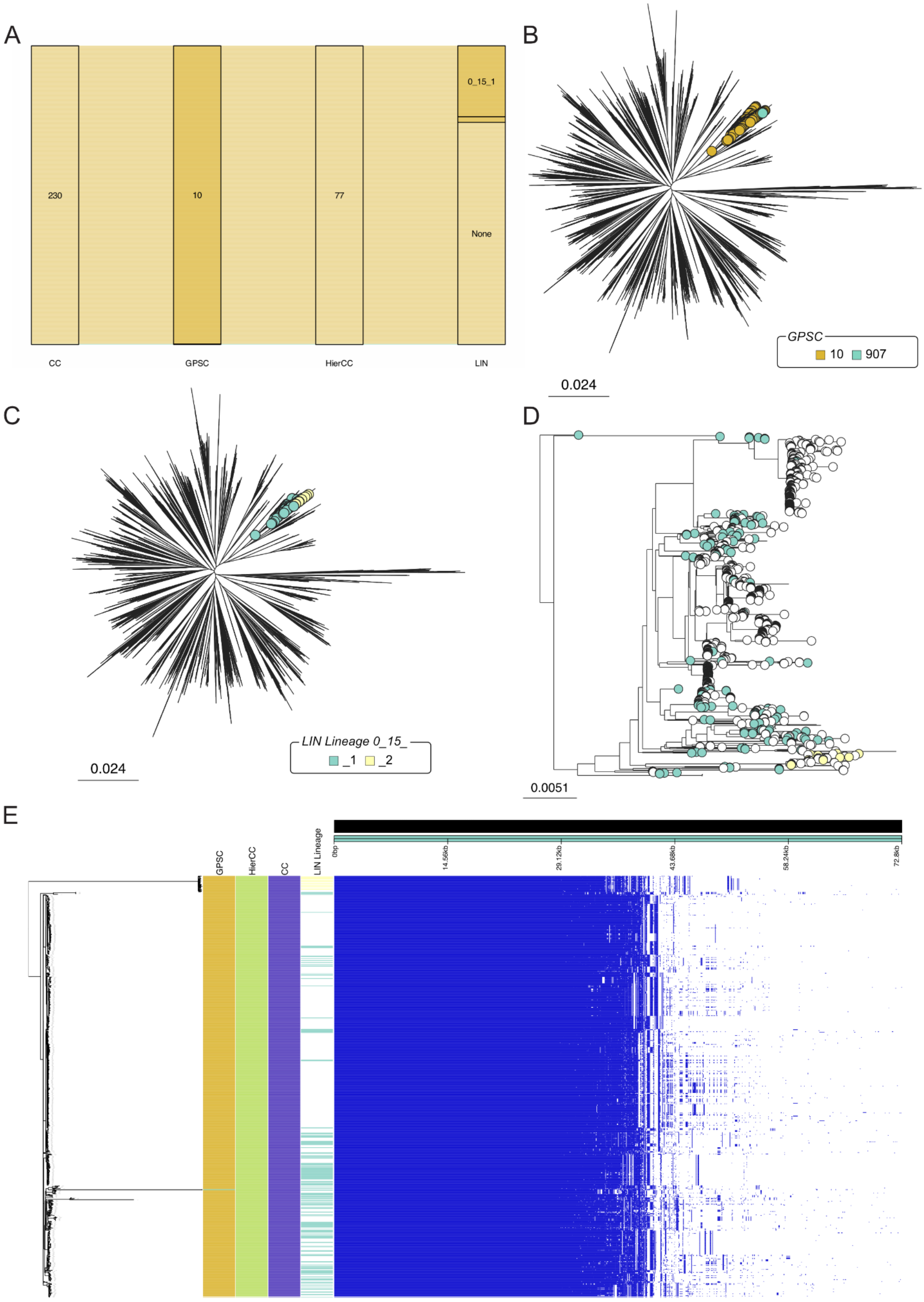

Supplementary Figure 3 - Analysis of genomes assigned to CC230. *(A) CC230 mostly consists of GPSC10, with two members of GPSC907. All genomes are members of HierCC 77. (B) In a species-wide phylogeny, the genomes cluster together. (C, D) The CC230 genomes consist of two LIN lineages, 0_15_1 and 0_15_2, with*

lineage 0_15_2 forming its own group. However, these lineages do not correspond to the GPSC lineages (E) Pan-genome analysis does not support the GPSC assignments, however there appear to be genomic differences between the LIN lineages.

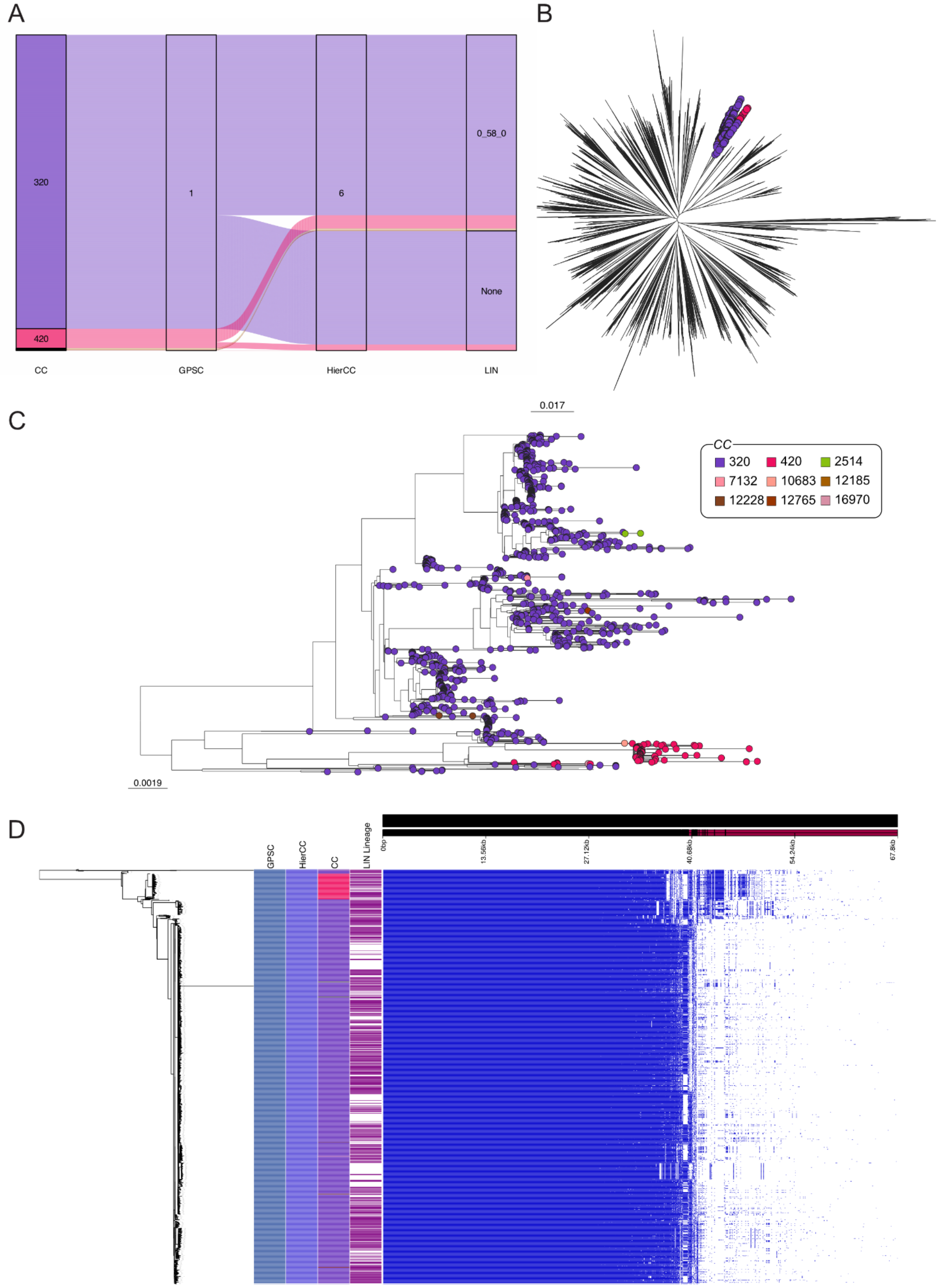

Supplementary Figure 4 - Analysis of genomes assigned to GPSC1. *(A) GPSC1 contains multiple CCs, but one LIN lineage and HierCC. (B) In a species-wide phylogeny, the genomes are in the same branch. (C) The*

subtree coloured by CC shows clustering of two dominant CCs, with many minor CCs scattered throughout. (D) Pan-genome analysis supports the separation of GPSC1 into separate clusters.

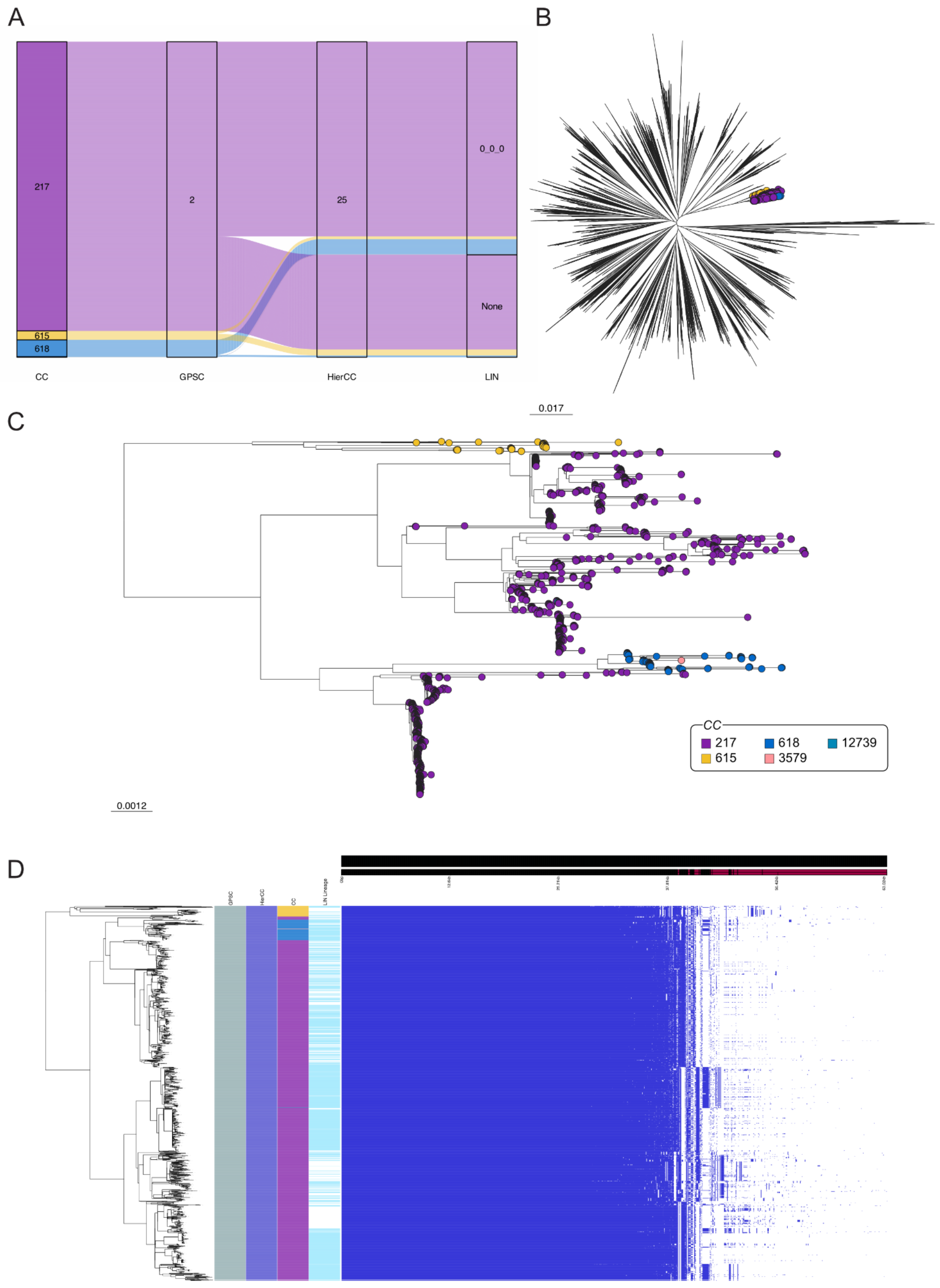

Supplementary Figure 5 - Analysis of genomes assigned to GPSC2. *(A) GPSC2 contains multiple CCs, but one LIN lineage and HierCC. (B) In a species-wise phylogeny, the genomes are in the same branch. (C) The*

subtree coloured by CC shows clustering of the main CCs, with minor CCs scattered throughout. (D) Pan-genome analysis supports the clustering of GPSC1 into a single group.

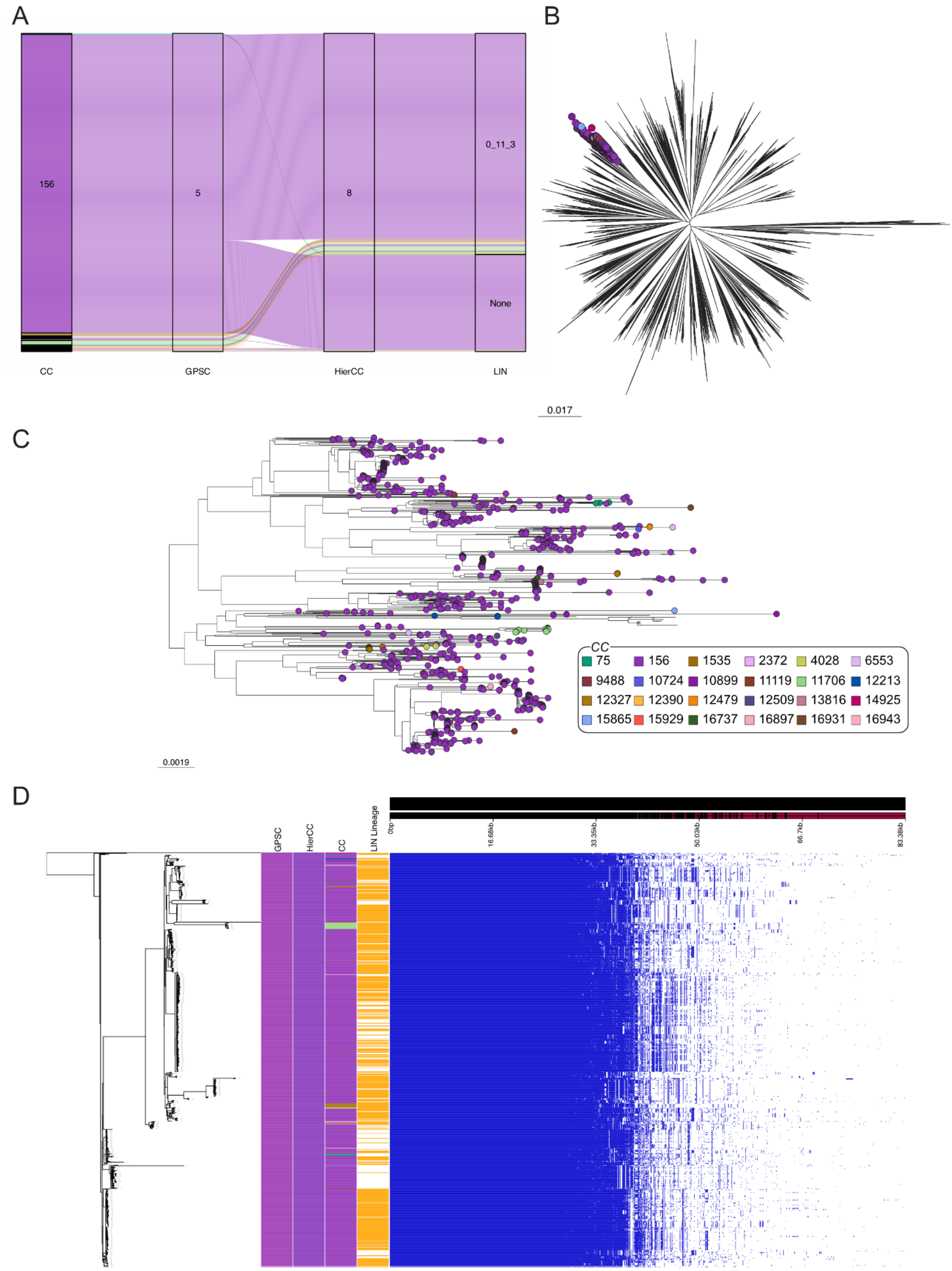

Supplementary Figure 6 - Analysis of genomes assigned to GPSC5. *(A) GPSC5 contains multiple CCs, but one LIN lineage and HierCC. (B) In a species-wide phylogeny, the genomes are in the same branch. (C) CC156 is dominant, with many others scattered throughout. (D) Pan-genome analysis supports the clustering of GPSC5 genomes into a single group, and does not provide support for the CC assignments.*

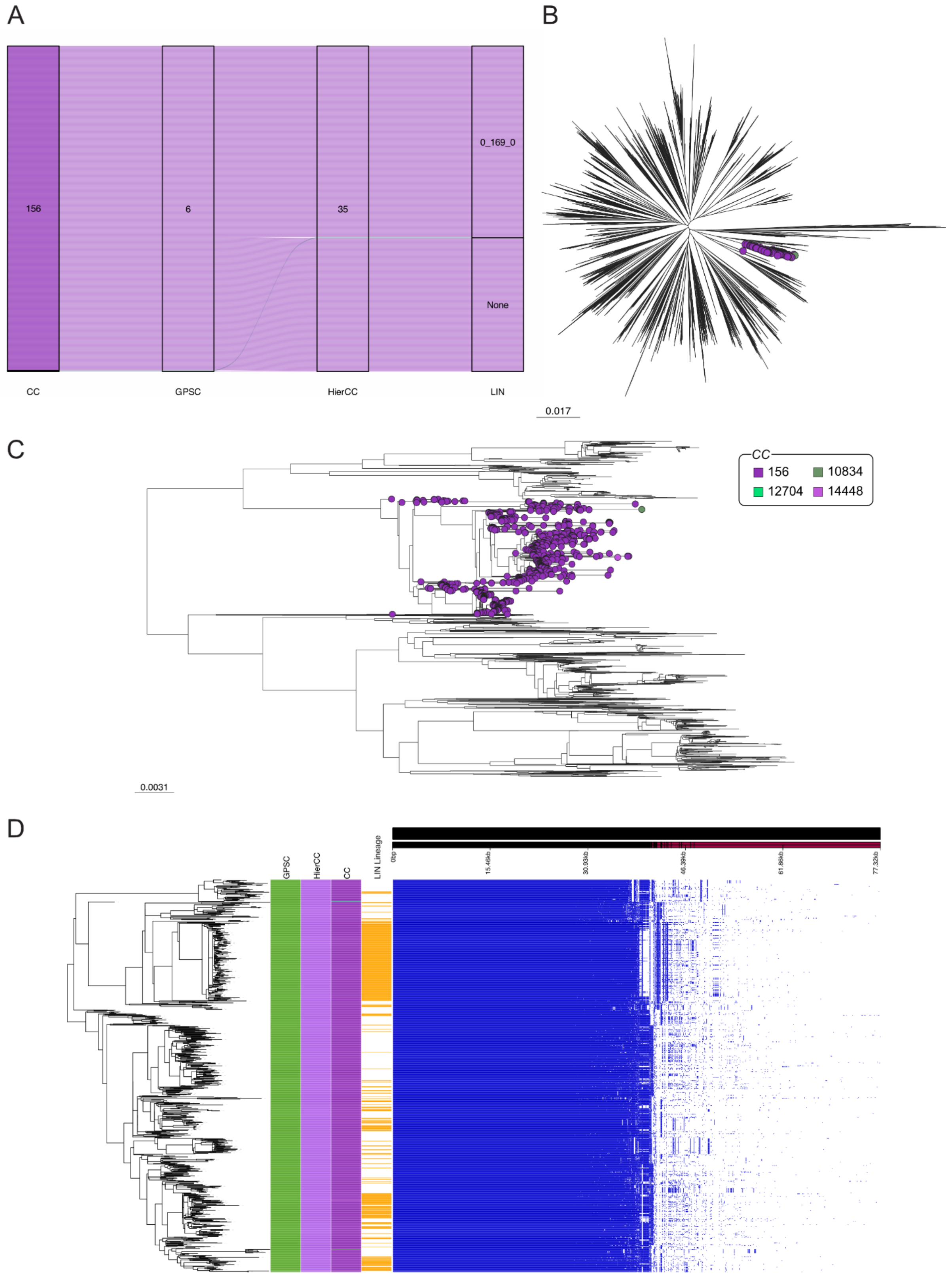

Supplementary Figure 7 - Analysis of genomes assigned to GPSC6. *(A) GPSC6 contains a single HierCC and*

LIN Lineage, but multiple minor CCs alongside the dominant CC156. (B) In a species-wide phylogeny, the genomes are in the same branch. (C) CC156 is dominant, with the other CCs spread across the tree. (D) Pan-genome analysis supports the clustering of GPSC6 genomes into one group.

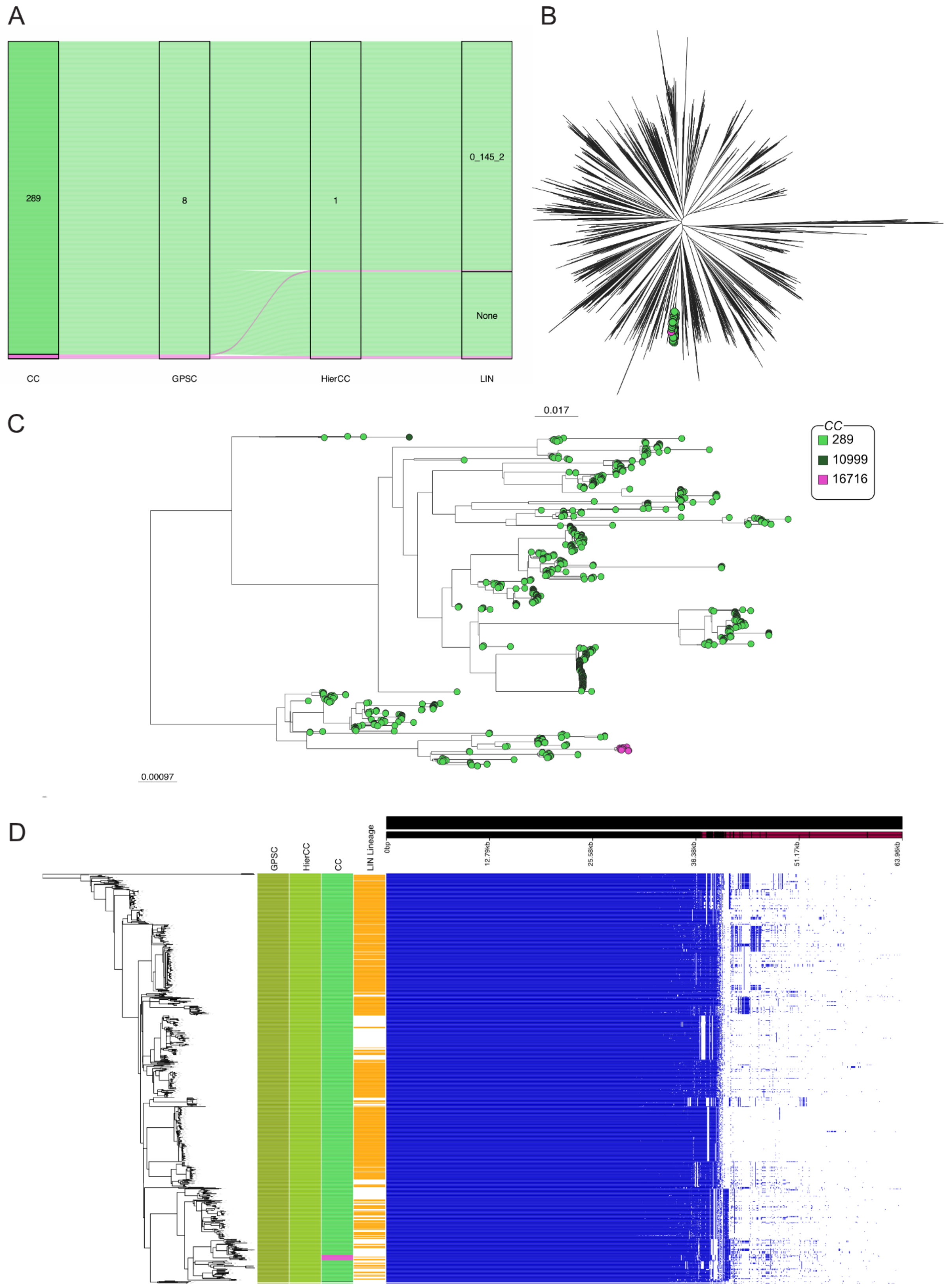

Supplementary Figure 8 - Analysis of genomes assigned to GPSC8. *(A) GPSC8 contains a single HierCC and LIN Lineage, but multiple CCs. (B) In a species-wide phylogeny, the genomes are in the same branch. (C) CC289 is dominant, with a small cluster of CC16716 genomes, and a single CC10999 genome. (D) Pan-genome analysis supports the clustering of GPSC6 genomes into one group, and does not support the multiple CCs.*

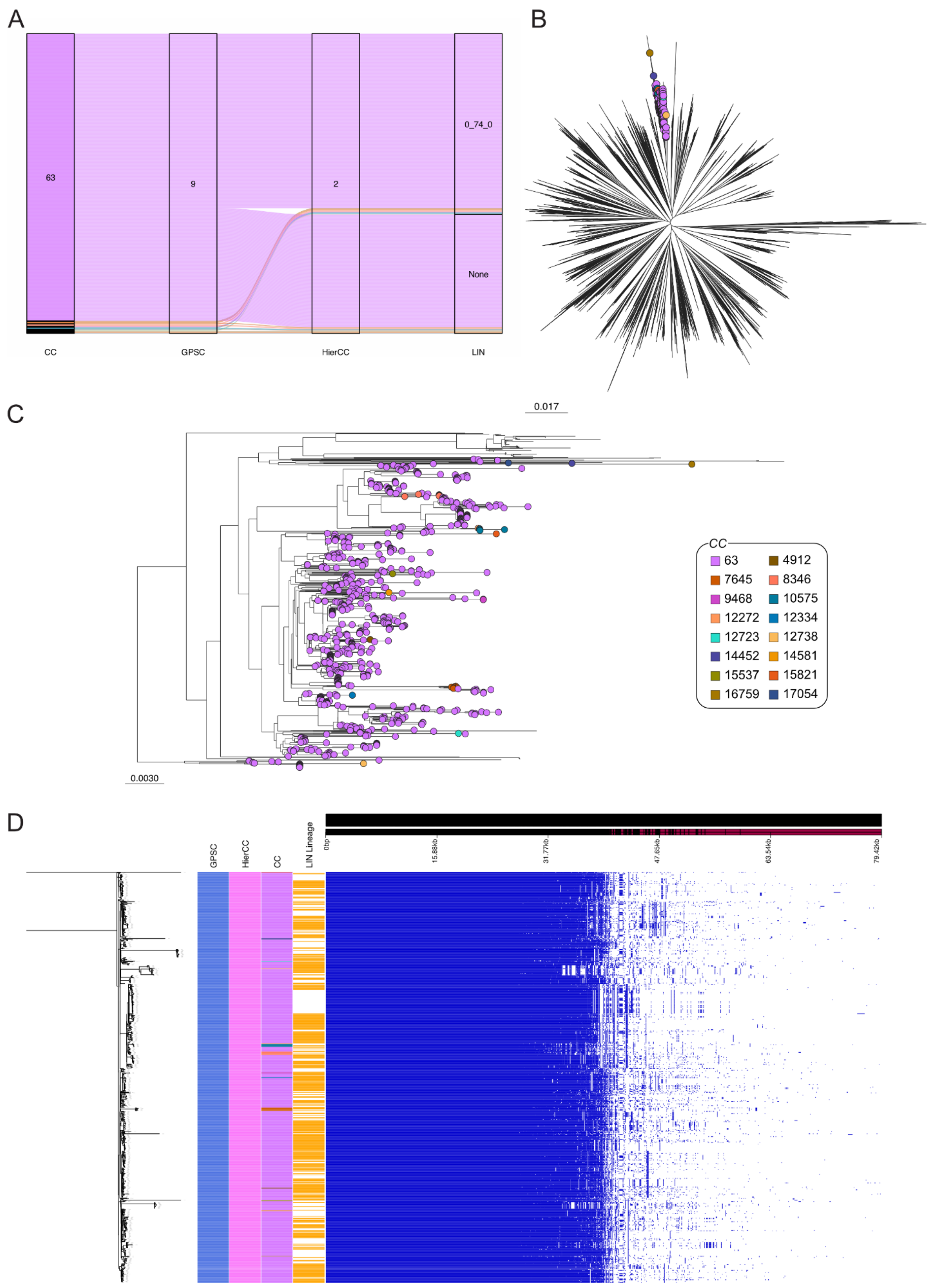

Supplementary Figure 9 - Analysis of genomes assigned to GPSC9. *(A) GPSC9 contains a single HierCC and LIN Lineage, but multiple CCs. (B) In a species-wide phylogeny, the genomes are in the same branch. (C) CC63 is dominant, but the phylogeny is very noisy with many minor CCs. (D) Pan-genome analysis supports the clustering*

of GPSC9 genomes into one group, and does not support the multiple CCs.

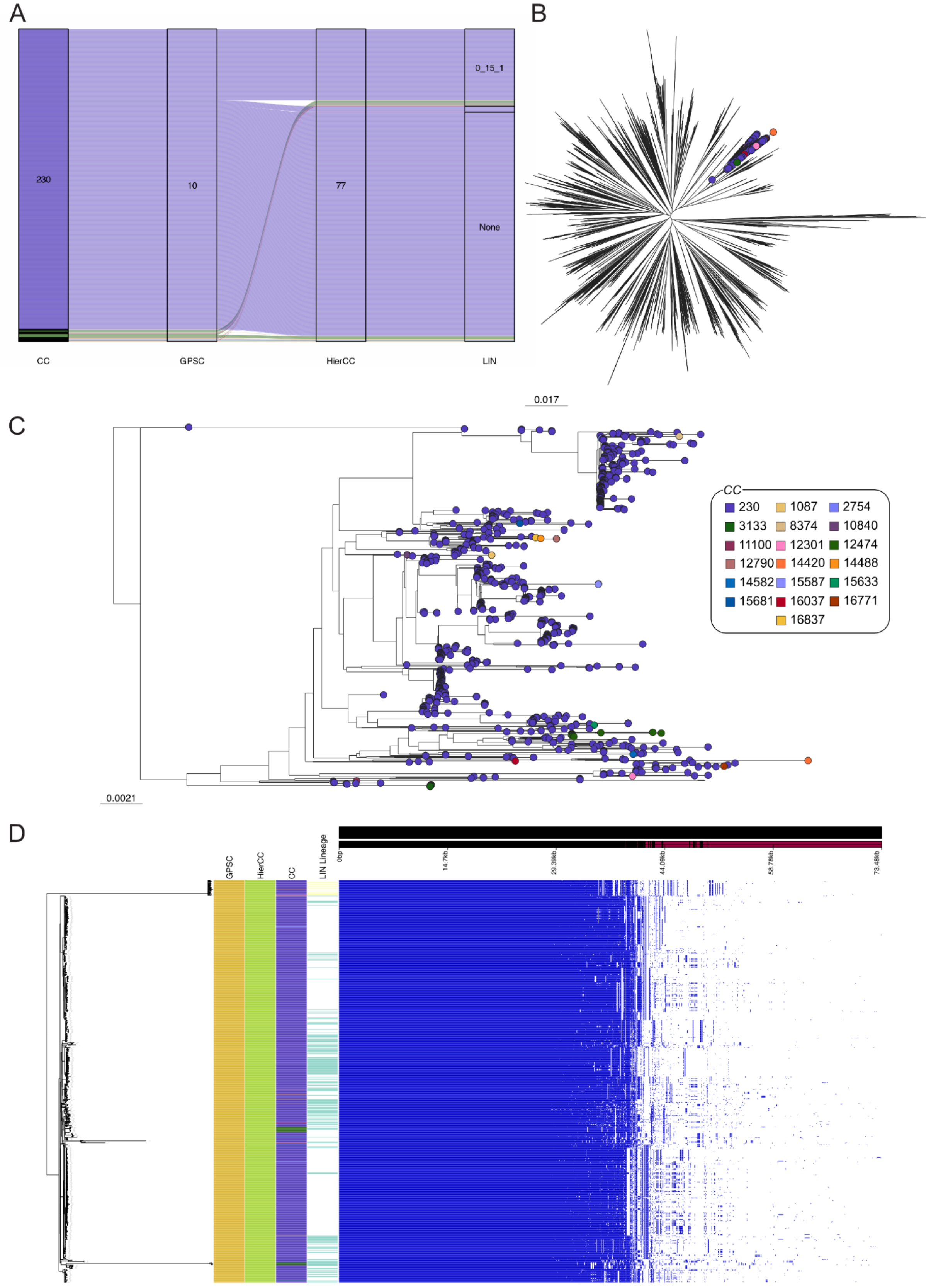

Supplementary Figure 10 - Analysis of genomes assigned to GPSC10. *(A) GPSC10 contains a single HierCC*

but multiple LIN lineages and CCs. (B) In a species-wide phylogeny, the genomes are in the same branch. (C) CC230 is dominant, but other CCs are spread throughout (D) Pan-genome analysis supports the clustering of GPSC10 genomes into one group, and does not support the multiple CCs. However, it does lend support for two the two identified LIN lineages.

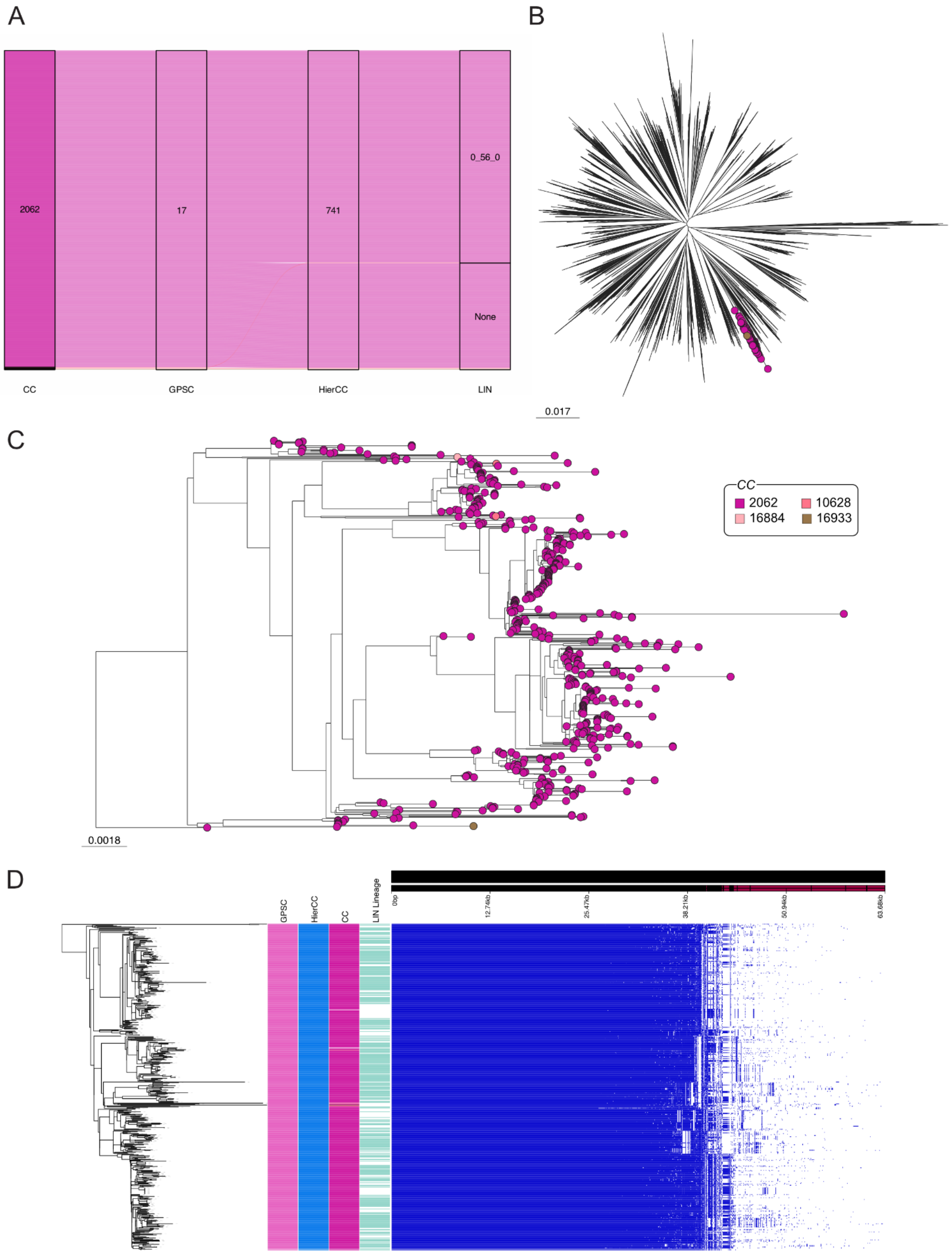

Supplementary Figure 11 - Analysis of genomes assigned to GPSC17. *(A) GPSC17 contains a single HierCC and LIN lineage, with four CCs. (B) In a species-wide phylogeny, the genomes are in the same branch. (C) CC2062 is dominant, with the other three represented by very few genomes. (D) Pan-genome analysis supports the*

clustering of GPSC17 genomes into one group, and does not support the multiple CCs.

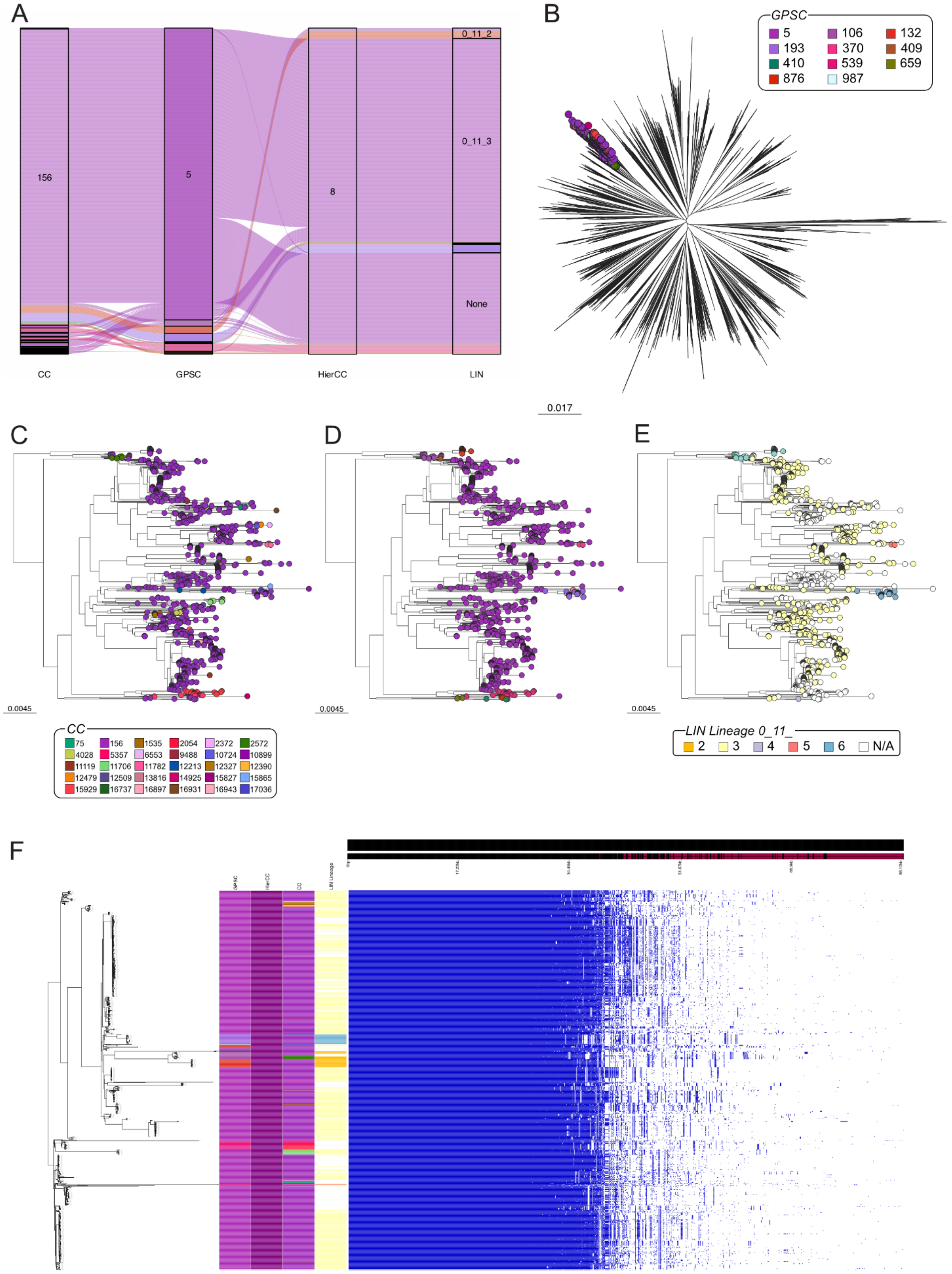

Supplementary Figure 12 - Analysis of genomes assigned to HierCC 8. *(A) HierCC 8 contains many GPSCs, CCs, and LIN lineages (B) In a species-wide phylogeny, the genomes are in the same branch. (C) CC156 is dominant, but other CCs are spread throughout and form small clusters (D) There is significant clustering based*

on GPSC and (E) LIN lineages. (F) Pan-genome analysis does not show huge genomic differences between genomes, however the branch lengths suggest that the division into separate clusters by GPSC assignments is a real effect.

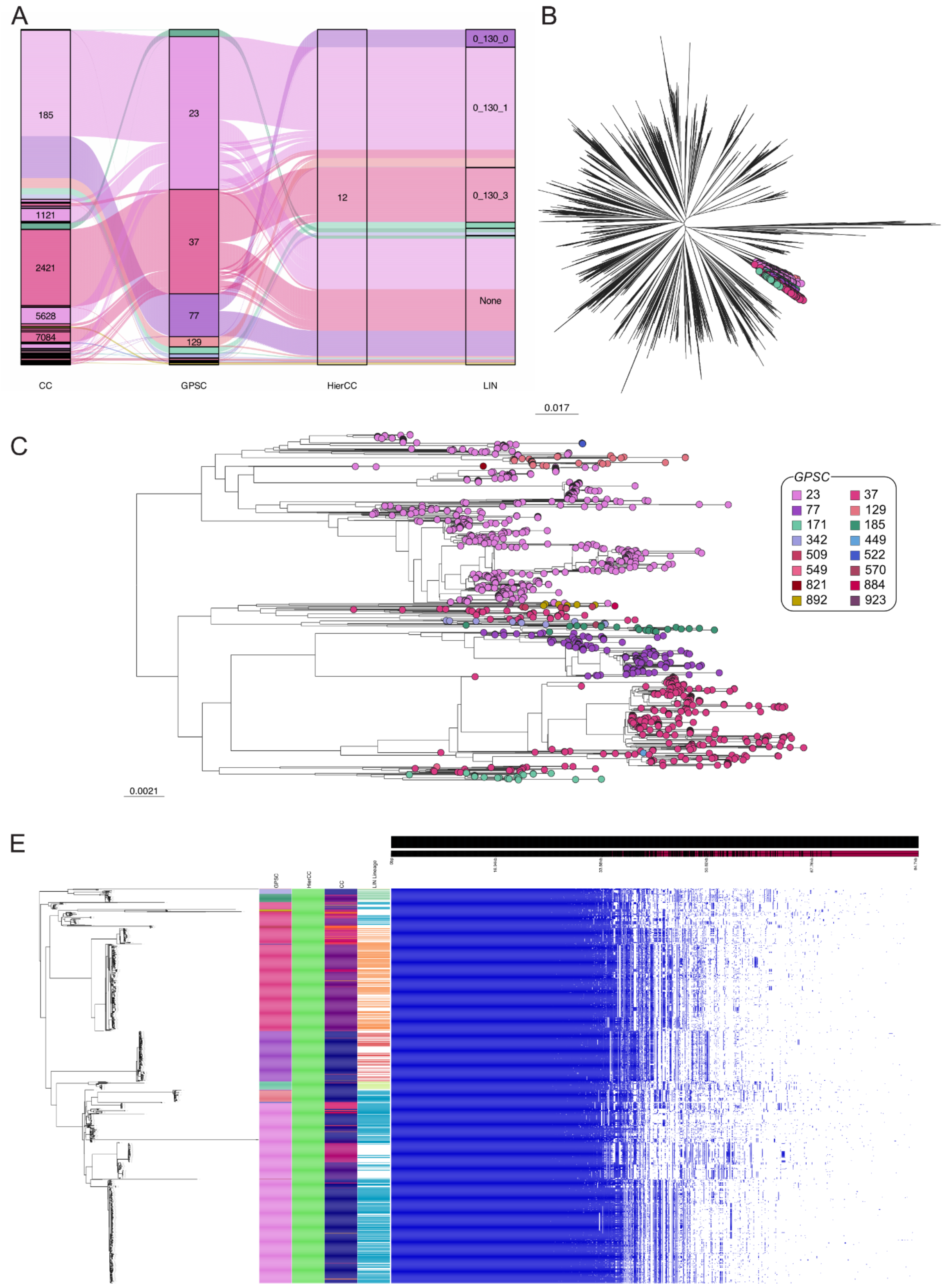

Supplementary Figure 13 - Analysis of genomes assigned to HierCC 12. *(A) HierCC 12 contains a huge number of GPSCs, CC, and LIN lineages. (B) In a species-wide phylogeny, the genomes are in the same branch. (C) Clear clustering is seen in the subtree based on GPSC. (D) Pan-genome analysis broadly supports the GPSC assignments, though there are still limited differences between the genomes.*

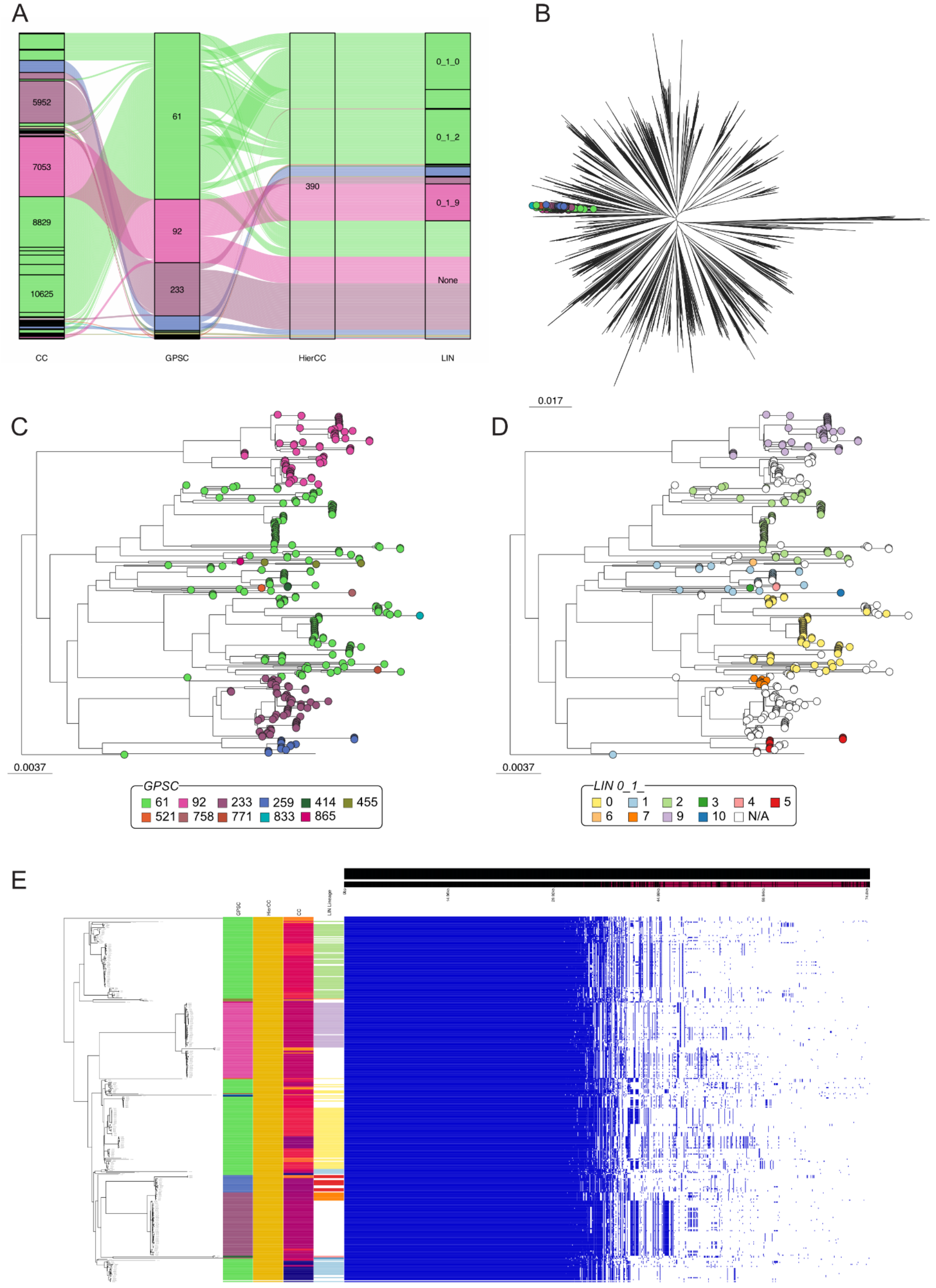

Supplementary Figure 14 - Analysis of genomes assigned to HierCC 390. *(A) HierCC 390 contains a huge*

number of GPSCs, CC, and LIN lineages. (B) In a species-wide phylogeny, the genomes are in the same branch. (C) Clear clustering is seen in the subtree based on GPSC and (D) LIN lineage. (E) Pan-genome analysis supports the GPSC assignments.

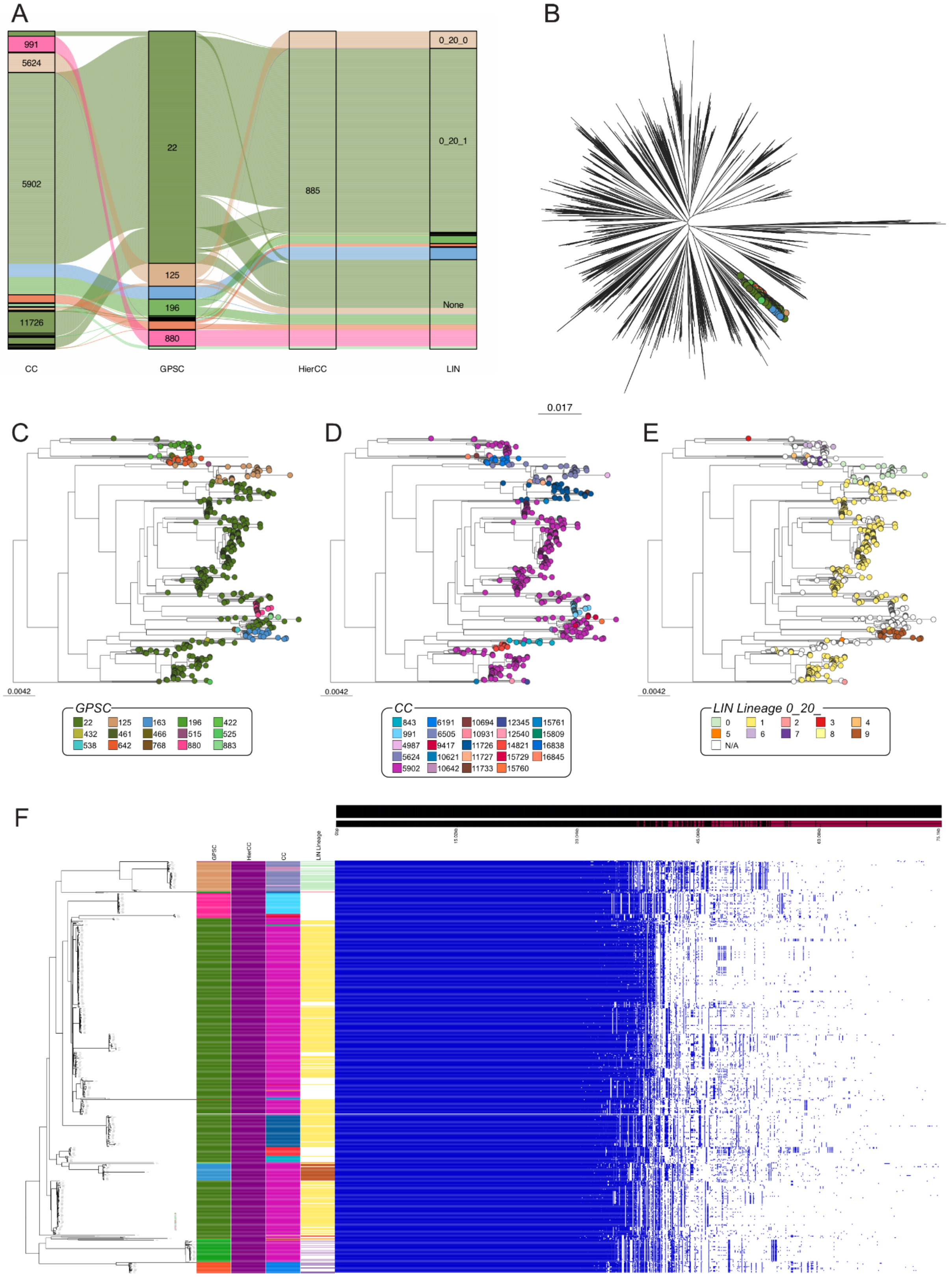

Supplementary Figure 15 - Analysis of genomes assigned to HierCC 885. *(A) HierCC 885 contains many GPSCs, CC, and LIN lineages. (B) In a species-wide phylogeny, the genomes are in the same branch. (C) Clear*

clustering is seen in the subtree based on GPSC, whereas (D) CC, and (E) LIN lineages do not cluster quite as well. (F) Pan-genome analysis supports the GPSC assignments, as seen by differences in the gene content.

Gene	*Non-unique Gene name*	*Annotation*
group_829		membrane protein
group_766		phage protein
group_1725		FIG01114970: hypothetical protein
group_1703		transposase;transposaseTransposase domain (DUF772)
group_1658		glutamate-cysteine ligase
group_1637		arsenate reductase
group_1630		site-specific recombinase;site-specific recombinaseRecombinase;site-specific recombinaseResolvase N terminal domain
group_1629		hypothetical protein
pezT_1	pezT_1	zeta toxinUDP-N-acetylglucosamine kinaseUncharacterized protein conserved in bacteriaZeta toxin
group_1517		FIG01114020: hypothetical proteinReplication initiator protein A (RepA) N-terminus
group_1513		transposase;transposaseTransposase DDE domain;transposaseTransposaseTransposase DDE domain
immR~~~immR_2~~~immR_1	immR;immR_2;immR_1	Cro/CI family transcriptional regulatorHTH-type transcriptional regulator immRPredicted transcriptional regulatorHelix-turn-helix
group_1500		ATPases with chaperone activity ATP-binding subunit
group_1490		Abortive infection protein AbiGIINucleotidyl transferase of unknown function (DUF1814)
group_1480		membrane protein
group_1478		FIG01114516: hypothetical protein
group_1429		FIG01116986: hypothetical proteinPrgI family protein
group_1408		Tn5252 Orf 10 protein
group_1386		Tn5253 hypothetical protein
group_1349		FIG01114502: hypothetical protein
group_1326		site-specific recombinasemultiple promoter invertaseResolvase N terminal domain
group_1312		Tn5252 ORF9Bacterial mobilisation protein (MobC)
group_1285		trsE-like proteinType IV secretory pathway VirB4 componentstype-IV secretion system protein TraCAAA-like domain
group_1278		hypothetical protein

group_782		hypothetical protein
hpaIIM_1	hpaIIM_1	C-5 cytosine-specific DNA methylaseModification methylase HpaIIDNA cytosine methylaseDNA (cytosine-5-)-methyltransferaseC-5 cytosine-specific DNA methylase
pezA	pezA	DNA-binding helix-turn-helix proteinAntitoxin PezAhypothetical proteinPredicted transcriptional regulatorputative zinc finger/helix-turn-helix protein YgiT familyHelix-turn-helix domain
group_692		hypothetical protein;Uncharacterized protein conserved in bacteria (DUF2326)
group_634		site-specific recombinaseRecombinase;site-specific recombinase
group_528		DNA primase (type)DNA primase (bacterial type)DNA primase;FIG01116415: hypothetical proteinDNA primase;DNA primase (type)
group_478		SNF2 family proteinDNA methylaseDNA phosphorothioation system restriction enzymeSNF2 family N-terminal domain;SNF2 family proteinDNA methylase
group_410		Tn5253 hypothetical protein
group_374		relaxaseRelaxase/Mobilisation nuclease domain
group_348		FIG01114872: hypothetical protein
ssp5	ssp5	Agglutinin receptorSSP-5Predicted outer membrane proteinadhesin isopeptide-forming adherence domainGlucan-binding protein C;Agglutinin receptorSSP-5adhesin isopeptide-forming adherence domainGlucan-binding protein C

Supplementary Table 1 - A selection of genes present in GPSC235, but not in GPSC9, despite both sets of genomes being clustered into HierCC2. *Taken from Panaroo annotation.*
